# Supplementary material for: Direct imaging of magnetotransport at graphene-metal interfaces with a single-spin quantum sensor
Source: Nat Commun. 2026 May 28;17:6913. doi: 10.1038/s41467-026-73480-8 (PMC13389063; doi:10.1038/s41467-026-73480-8)
Supplement: Supplementary file 1 — Supplementary Information [file 41467_2026_73480_MOESM1_ESM.pdf]

Supplementary Information:
Direct imaging of magnetotransport at graphene-metal interfaces with a single-spin quantum sensor

C. Ding[1], M. L. Palm[1], K. Kohli[1], T. Taniguchi[2], K. Watanabe[3], C. L. Degen[1,4]

[1]*Department of Physics, ETH Zürich, Otto Stern Weg 1, 8093 Zürich, Switzerland.*

[2]*Research Center for Materials Nanoarchitectonics, National Institute for Materials Science, 1-1 Namiki, Tsukuba 305-0044, Japan.*

[3]*Research Center for Electronic and Optical Materials, National Institute for Materials Science, 1-1 Namiki, Tsukuba 305-0044, Japan. and*

[4]*Quantum Center, ETH Zürich, 8093 Zürich, Switzerland.**

(Dated: March 20, 2026)

1. THEORY OF TWO-CARRIER MODEL AND MAGNETORESISTANCE (MR) IN GRAPHENE

In the (semi-)classical formalism, the equation of motion of electrons and holes in graphene under electric ($\mathbf{E}$) and magnetic ($\mathbf{B}$) fields can be described by the following equations [1],

$$\begin{cases} -e(\mathbf{E}+\mathbf{v}_\mathrm{e}\times\mathbf{B})/m_\mathrm{e} = (\mathbf{v}_\mathrm{e}-\mathbf{v}_\mathrm{h})/(2\tau_\mathrm{eh}) + \mathbf{v}_\mathrm{e}/\tau_{e-\mathrm{mr}} \ , \\ e(\mathbf{E}+\mathbf{v}_\mathrm{h}\times\mathbf{B})/m_\mathrm{h} = -(\mathbf{v}_\mathrm{e}-\mathbf{v}_\mathrm{h})/(2\tau_\mathrm{eh}) + \mathbf{v}_\mathrm{h}/\tau_{h-\mathrm{mr}} \ , \end{cases} \tag{S1}$$

where $\mathbf{v}_\mathrm{e/h}$ are the drift velocities of electrons/holes, $m_\mathrm{e/h}$ their effective masses, $\tau_\mathrm{e/h-mr}$ the momentum-relaxing times, and τ_eh the electron-hole scattering time. Consequently, the total scattering time is $\tau_\mathrm{e/h} = (\tau_\mathrm{eh}^{-1} + \tau_\mathrm{e/h-mr}^{-1})^{-1}$. In the following, electron-hole symmetry ($m_\mathrm{e} = m_\mathrm{h} = m$ and $\tau_\mathrm{e} = \tau_\mathrm{h} = \tau$) is assumed.

In the case of an out-of-plane magnetic field $\mathbf{B} = B_\mathrm{ext}\hat{\mathbf{z}}$ and an in-plane electrical field $\mathbf{E}$, the drift velocities can be expressed as [1]

$$\mathbf{v}_\mathrm{e/h} = (\mp\mu_0\mathbf{E} + \mu_B^2 B_\mathrm{ext}\mathbf{E}\times\hat{\mathbf{z}})/(1+(\mu_B B_\mathrm{ext})^2) \ , \tag{S2}$$

where $\mu_0 = e\tau/m$ is the zero-field mobility and $\mu_B = e\sqrt{\tau\tau_\mathrm{mr}}/m$ the magnetotransport mobility. The ratio between the two is given by $\mu_B/\mu_0 = \sqrt{1+\tau_\mathrm{mr}/\tau_\mathrm{eh}} \geq 1$.

Note that the τ_eh term contributes only when electrons and holes co-exist (*i.e.*, around charge neutrality) and strongly interact. This can lead to a μ_B/μ_0 enhancement of up to $\sim 3\times$ [1]. In the single-carrier regime or for non-interacting electron-hole puddles [2], $\tau_\mathrm{mr}/\tau_\mathrm{eh} = 0$ and $\mu_B = \mu_0$.

The electron/hole current densities are related to the drift velocities via

$$\mathbf{J}_\mathrm{e/h} = \mp e n_\mathrm{e/h}\mathbf{v}_\mathrm{e/h} \ , \tag{S3}$$

where $n_\mathrm{e/h}$ are the carrier density of electrons/holes. The resulting total current density is

$$\mathbf{J} = \mathbf{J}_\mathrm{e} + \mathbf{J}_\mathrm{h} = \sigma_{xx}\mathbf{E} + \sigma_{xy}\mathbf{E}\times\hat{\mathbf{z}} \ , \tag{S4}$$

where

$$\begin{cases} \sigma_{xx} = e\mu_0(n_\mathrm{e}+n_\mathrm{h})/(1+(\mu_B B_\mathrm{ext})^2) \\ \sigma_{xy} = e\mu_B(n_\mathrm{h}-n_\mathrm{e})\mu_B B_\mathrm{ext}/(1+(\mu_B B_\mathrm{ext})^2) \end{cases} . \tag{S5}$$

* degenc@ethz.ch

The longitudinal resistivity and the corresponding (relative) magnetoresistivity are obtained as

$$\rho_{xx} = \frac{\sigma_{xx}}{\sigma_{xx}^2 + \sigma_{xy}^2} = \frac{1}{e(n_\mathrm{e} + n_\mathrm{h})\mu_0} \frac{1 + (\mu_B B_\mathrm{ext})^2}{1 + p^2(\frac{\mu_B}{\mu_0})^2(\mu_B B_\mathrm{ext})^2} \ , \tag{S6}$$

$$\mathrm{MR}_{xx} = \frac{\rho_{xx}(B_\mathrm{ext}) - \rho_{xx}(B_\mathrm{ext} = 0)}{\rho_{xx}(B_\mathrm{ext} = 0)} = \frac{1 - p^2(\mu_B/\mu_0)^2}{1 + p^2(\mu_B/\mu_0)^2(\mu_B B_\mathrm{ext})^2}(\mu_B B_\mathrm{ext})^2 \ , \tag{S7}$$

where $p = (n_\mathrm{e} - n_\mathrm{h})/(n_\mathrm{e} + n_\mathrm{h}) \in [-1, 1]$ characterizes the imbalance between electron and hole densities.

For graphene at the charge-neutrality point (CNP), where the electron density is equal to the hole density, $p = 0$ and therefore $\mathrm{MR}_{xx} = (\mu_B B_\mathrm{ext})^2$. Depending on the electron-hole interacting strength, μ_B can vary from μ_0 to around $3\mu_0$ [1]. By contrast, in highly-doped graphene where only one carrier type exists, $p^2 = 1$ and $\mu_B = \mu_0$, which result in $\rho_{xx} = 1/(en_\mathrm{e/h}\mu_0)$ and $\mathrm{MR}_{xx} = 0$, recovering the single-carrier model. As for the transition between the two extreme cases, it can be expected that increasing the imbalance of the carrier doping would decrease μ_B towards μ_0 and reduce MR_{xx} towards zero at a finite B_ext.

According to band theory, the carrier densities of electrons and holes in graphene can be calculated as

$$\begin{cases} n_\mathrm{e} = \int_0^\infty f_\mathrm{FD}(E)D(E)dE \\ n_\mathrm{h} = \int_{-\infty}^0 (1 - f_\mathrm{FD}(E))D(E)dE \end{cases} , \tag{S8}$$

where $f_\mathrm{FD}(E) = 1/(e^{\frac{E-E_\mathrm{F}}{k_\mathrm{B}T}} + 1)$ is the Fermi-Dirac distribution, E_F the Fermi energy, and $D(E)$ the density of states. For monolayer graphene (MLG), $D(E) = \frac{2|E|}{\pi(\hbar v_\mathrm{F})^2}$. By substituting E with $x(E) = (E - E_\mathrm{F})/(k_\mathrm{B}T)$, one obtains

$$\begin{cases} n_\mathrm{e} = \frac{2(k_\mathrm{B}T)^2}{\pi(\hbar v_\mathrm{F})^2}[\int_{x(0)}^\infty \frac{x}{e^x+1}dx - x(0)\int_{x(0)}^\infty \frac{1}{e^x+1}dx] \\ n_\mathrm{h} = \frac{2(k_\mathrm{B}T)^2}{\pi(\hbar v_\mathrm{F})^2}[\int_{-x(0)}^\infty \frac{x}{e^x+1}dx + x(0)\int_{-x(0)}^\infty \frac{1}{e^x+1}dx] \end{cases} , \tag{S9}$$

where the first and second integrals are mainly contributed by the internal carriers (*e.g.*, from thermal excitation or residue charge inhomogeneities) and the external carriers (*e.g.*, from the electrostatic gating), respectively.

At the CNP, $E_\mathrm{F} = 0$, Eqn. S9 results in equal electron and hole densities of

$$n_T = \frac{2\pi^3}{3}\left(\frac{k_\mathrm{B}T}{hv_\mathrm{F}}\right)^2 \tag{S10}$$

For room temperature $T = 300$ K, $n_T \approx 0.08 \times 10^{12}$ cm^{-2}.

Away from CNP, $|E_\mathrm{F}| > 0$, which corresponds to imbalanced electron and hole densities. The net carrier density relative to the CNP is characterized by $n = n_\mathrm{e} - n_\mathrm{h}$. In the extreme case where $|E_\mathrm{F}| \gg k_\mathrm{B}T$, $f_\mathrm{FD}(E)$ becomes a unit step function at $E = E_\mathrm{F}$, resulting in $(n_\mathrm{e}, n_\mathrm{h}) = (\frac{E_\mathrm{F}^2}{\pi(\hbar v_\mathrm{F})^2}, 0)$ for $E_\mathrm{F} > 0$ and $(n_\mathrm{e}, n_\mathrm{h}) = (0, \frac{E_\mathrm{F}^2}{\pi(\hbar v_\mathrm{F})^2})$ for $E_\mathrm{F} < 0$, which recovers the typical single-carrier formalism in highly-doped graphene.

2. FINITE ELEMENT SIMULATIONS OF CURRENT DISTRIBUTIONS

Simulations of two-dimensional current distribution $\mathbf{J}(x, y)$ are performed with the finite element method using the *Coefficient PDE* module of COMSOL Multiphysics, following previous work [3, 4]. The macroscopic semi-classical magnetotransport model is described by the following two equations (Methods and [5]),

$$-\sigma \boldsymbol{\nabla} \Phi(\mathbf{r}) = (1 - D_\nu^2 \boldsymbol{\nabla}^2)\mathbf{J}(\mathbf{r}) + \mu B_\mathrm{ext}(1 + D_H^2 \boldsymbol{\nabla}^2)\mathbf{J}(\mathbf{r}) \times \hat{\mathbf{z}} \ , \tag{S11}$$

$$\boldsymbol{\nabla} \cdot \mathbf{J}(\mathbf{r}) = 0 \ , \tag{S12}$$

They can be combined in the following form, resulting in the central differential equations to be solved,

$$\boldsymbol{\nabla} \cdot (-c\boldsymbol{\nabla}\mathbf{u} - \alpha\mathbf{u} + \gamma) + \beta \cdot \boldsymbol{\nabla}\mathbf{u} + a\mathbf{u} = f \ , \tag{S13}$$

where $\mathbf{u}(x, y) = (\Phi(x, y), J_x(x, y), J_y(x, y))^\mathrm{T}$. For graphene, the coefficients $\alpha_\mathrm{Gr} = \gamma_\mathrm{Gr} = f_\mathrm{Gr} = 0$, and others are defined as:

$$c_\mathrm{Gr} = \begin{pmatrix} 0 & 0 & 0 \\ 0 & D_\nu^2 & -\mu_\mathrm{Gr} B_\mathrm{ext} D_H^2 \\ 0 & \mu_\mathrm{Gr} B_\mathrm{ext} D_H^2 & D_\nu^2 \end{pmatrix} \tag{S14}$$

$$\beta_\mathrm{Gr} = \begin{pmatrix} \begin{pmatrix} 0 & 0 \end{pmatrix} & \begin{pmatrix} 1 & 0 \end{pmatrix} & \begin{pmatrix} 0 & 1 \end{pmatrix} \\ \begin{pmatrix} \sigma_\mathrm{Gr} & 0 \end{pmatrix} & \begin{pmatrix} 0 & 0 \end{pmatrix} & \begin{pmatrix} 0 & 0 \end{pmatrix} \\ \begin{pmatrix} 0 & \sigma_\mathrm{Gr} \end{pmatrix} & \begin{pmatrix} 0 & 0 \end{pmatrix} & \begin{pmatrix} 0 & 0 \end{pmatrix} \end{pmatrix} \tag{S15}$$

$$a_\mathrm{Gr} = \begin{pmatrix} 0 & 0 & 0 \\ 0 & 1 & \mu_\mathrm{Gr} B_\mathrm{ext} \\ 0 & -\mu_\mathrm{Gr} B_\mathrm{ext} & 1 \end{pmatrix} \tag{S16}$$

where c_{Gr} corresponds to the hydrodynamic terms in the model. The mobility and conductivity of graphene,

$$\mu_{\text{Gr}} = \frac{n_{\text{e}} - n_{\text{h}}}{n_{\text{e}} + n_{\text{h}}} \frac{\mu_B^2}{\mu_0} \ , \tag{S17}$$

$$\sigma_{\text{Gr}} = e\mu_0(n_{\text{e}} + n_{\text{h}}) \frac{1 + (\mu_{\text{Gr}} B_{\text{ext}})^2}{1 + (\mu_B B_{\text{ext}})^2} \ , \tag{S18}$$

are defined to be consistent with the two-carrier diffusive model shown in Eqn. S5. We assume electron-hole symmetry [1].

As discussed in Section 1, strong electron-hole scattering (relative to the momentum-relaxing scattering) can result in μ_B decreasing from up to $\sim 3\mu_0$ at $n = 0$ to μ_0 at high n, while the exact dependence (on n) is not clear and can also vary among devices. For simplicity, we assume $\mu_B = \mu_0$ for all n in the simulation, which corresponds to the case of weak electron-hole scattering around the CNP ($\tau_{\text{eh}} \gg \tau_{\text{mr}}$). As will be shown, this simplified consideration can reproduce the MR effect in graphene reasonably well.

Regarding the boundary setting, we impose Dirichlet boundary conditions that fix the electrical potential at source and drain contacts, specifically $\Phi_S = V_{\text{SD}}$ and $\Phi_D = 0$. In addition, a no-slip boundary condition fixing the current $J_x = J_y = 0$ is imposed for the remaining boundaries of graphene.

2.1. Simulation of a graphene channel and verification of the two-carrier model

The implementation of the two-carrier diffusive model of graphene is verified by simulating the channel geometry (with width W and length L) and extracting the two-terminal resistance R as a function of (B_{ext}, n), which is then compared against analytical formulas.

Regarding the input parameters for COMSOL (Eqn. S13), the mobility is set to $\mu_0 = \mu_B = 2\ \text{m}^2/(\text{V} \cdot \text{s})$. The hydrodynamic terms are neglected by setting them to $D_\nu = D_H = 1\,\text{nm}$. For the carrier density dependence, we set $n_T \approx 0.08 \times 10^{12}\ \text{cm}^{-2}$ corresponding to room temperature ($T = 300$ K) and sweep E_{F} from $-4k_{\text{B}}T$ to $4k_{\text{B}}T$. For each E_{F}, $n_{\text{e/h}}$ (and accordingly $n = n_{\text{e}} - n_{\text{h}}$) can be calculated through Eqn. S9. The two-terminal resistance can be obtained through $R = V_{\text{SD}}/I$, where the source-drain voltage $V_{\text{SD}} = 0.2$ V and I is calculated by integrating the simulated $J_n = \mathbf{J} \cdot \mathbf{n}$ across the channel.

Fig. S9a shows R as a function of n for different B_{ext}. The curves correspond to the analytical results $R = g\rho_{xx}$, where ρ_{xx} is calculated using Eqn. S6 and g is a geometric

factor. (For the channel geometry with $W = 1.5$ μm and $L = 6$ μm, $g = L/W = 4$). The dots correspond to the numerical results from COMSOL, which match the analytical curves reasonably well. As expected, R increases the fastest with B_{ext} at the CNP ($n = 0$). When n_{e} and n_{h} become more imbalanced, the increase of R with B_{ext} becomes less prominent. Fig. S9b shows MR at the CNP as a function of B_{ext}, where both analytical (small dots) and numerical (large dots) results align perfectly with the trend $MR \propto (\mu_B B_{\mathrm{ext}})^2$ (gray curve).

2.2. Modeling and simulation of the graphene-metal hybrid device

The hybrid device we investigate contains regions of graphene and metal (Cr/Au). Additionally, to account for the contact resistance, a graphene-metal interface region is included [6].

The graphene is modeled with the two-carrier description discussed in Section 1. For the metal (M) and the circular contact (C) region, a conventional diffusive (ohmic) model is used, corresponding to $c_{\mathrm{M/C}} = \alpha_{\mathrm{M/C}} = \gamma_{\mathrm{M/C}} = f_{\mathrm{M/C}} = 0$ and

$$\beta_{\mathrm{M/C}} = \begin{pmatrix} \begin{pmatrix} 0 & 0 \end{pmatrix} & \begin{pmatrix} 1 & 0 \end{pmatrix} & \begin{pmatrix} 0 & 1 \end{pmatrix} \\ \begin{pmatrix} \sigma_{\mathrm{M/C}} & 0 \end{pmatrix} & \begin{pmatrix} 0 & 0 \end{pmatrix} & \begin{pmatrix} 0 & 0 \end{pmatrix} \\ \begin{pmatrix} 0 & \sigma_{\mathrm{M/C}} \end{pmatrix} & \begin{pmatrix} 0 & 0 \end{pmatrix} & \begin{pmatrix} 0 & 0 \end{pmatrix} \end{pmatrix}, a_{\mathrm{M/C}} = \begin{pmatrix} 0 & 0 & 0 \\ 0 & 1 & 0 \\ 0 & 0 & 1 \end{pmatrix}, \tag{S19}$$

where $\sigma_{\mathrm{M/C}}$ represents the conductivity in the corresponding region. Note that due to the low carrier mobility in the metal (on the order of 10^{-4} $\mathrm{m^2/V/s}$) and even lower value for contact regions, $\mu_{\mathrm{M/C}} B_{\mathrm{ext}} \ll 1$ and is therefore neglected in the matrix coefficients. At the boundary between different regions, the continuity equation is maintained.

The model of the device geometry, specifically the allocation of graphene and metal disc in the device, is based on the initial design and the AFM image (Fig. 1d). The circular graphene-metal interface (with width t) is divided into two sub-regions with low and high contact resistances ($R_{\mathrm{C1/2}} = t/\sigma_{\mathrm{C1/2}}$), parameterized by two angles (α, β) as shown in Fig. S10. The low-R_{C} region lets current pass smoothly while the high-R_{C} region hinders current flow. The necessity of including such non-uniform interface resistances in our model is demonstrated in Fig. S15 and will be discussed later.

To obtain good agreement between simulated current density maps with all our measurements (over about 9 months), we need to model three device states ($\mathrm{s}_{0,1,2}$) with different

parameters of the graphene-metal interface region (e.g. α and β). s_0 reflects the initial state, which can account for all measurements with R-T, R-V and Q-V configurations, while s_1 and s_2 have an increasingly extended graphene-metal interface region through which current is hindered. This suggests twice irreversible degradations of graphene-metal interfaces in our device. We also note that the degradations did not happen during the measurement periods but were observed after certain changes of the setup (e.g. re-wirebonding of the device), and therefore they could be caused by large transient currents passing through the device during these changes. In addition, a current leakage through the top left contact S is also included in s_1 and s_2 to match simulations with the measurements.

A summary of the geometric parameters and corresponding values of the device model is given in Table S1.

The main material parameters of the model are listed in Table S2. To estimate relevant parameters of the model for reproducing the measurement results, information from both bulk electrical characterization and local current imaging is used. Specifically, the bulk resistance characterization (see Fig. S8) shows that the CNP (corresponding to the maximal resistance) is reached at $V_{\mathrm{BG}}^{\mathrm{CNP}} \approx 0.175$ V. Accordingly, for a given V_{BG}, the net carrier density n relative to CNP can be obtained through the capacitance model (see Methods). This can be further used to calculate E_{F} together with $n_{\mathrm{e/h}}$ based on Eqn. S9, where the temperature is set as $T = 300$ K to reflect our measurement condition. For example, at $V_{\mathrm{BG}} = 0$, $n \approx -0.14 \times 10^{12}$ cm^{-2}, which corresponds to $E_{\mathrm{F}} \approx -0.974\ k_{\mathrm{B}}T$ with $(n_{\mathrm{e}}, n_{\mathrm{h}}) \approx (0.034, 0.174) \times 10^{12}$ cm^{-2}.

Next, the carrier (zero-field) mobility μ_0 can be extracted independently from the Hall angle deflection $\tan|\theta_H| = \mu_{\mathrm{Gr}} B_{\mathrm{ext}}$ (see the main text) and the bulk resistance measurements (see Methods). We note that the estimates from the two methods agree well. For all simulations shown in the main text, we use $\mu_0 = 1.4$ m^2/V/s, corresponding to a conductivity of graphene $\sigma_{\mathrm{Gr}} = 4.7 \times 10^{-4}$ S at $V_{\mathrm{BG}} = 0$ based on Eqn. S17.

The conductivity of the metal disc σ_{M} can be obtained according to the relative current flow through the metal disc and the graphene ring at different carrier densities. Specifically, as can be seen from the current distribution in the bottom right of the graphene-metal interface where the contact resistance is low (Fig. S16 for instance), the current flows more through the graphene ring at higher doping while entering into the metal disc more around the CNP. This can be attributed to the increase of graphene conductivity with progressive

electron or hole doping. In other words, when the conductivity of graphene σ_{Gr} surpasses σ_{M}, more current will flow through the graphene ring. Therefore, by combining the measured current distribution images at various carrier doping levels and the corresponding calculated σ_{Gr}, the value of σ_{M} can be estimated.

The conductivity of the high R_{C} region (thick red contour in Fig. S10) $\sigma_{\mathrm{C1}} = t/R_{\mathrm{C1}}$ is set to a low enough value such that the current is blocked to go through and the transport behavior does not change anymore when further decreasing the value (which is independent of whether the graphene is in the diffusive or hydrodynamic regime). Regarding the low R_{C} region (thin green contour in Fig. S10), since the current could pass this interface smoothly at $V_{\mathrm{BG}} = 0$, we assumed a smooth graphene-metal transition in this region without considering an additional interface resistance, which was also equivalent to setting $\sigma_{\mathrm{C2}} = t/R_{\mathrm{C2}}$ close to σ_{Gr} at $V_{\mathrm{BG}} = 0$. We note that in the device state s_2, however, σ_{C2} needed to be lower to reproduce the last set of measurements with P-R configuration (Fig. S18) where the current flowed more through the graphene ring even around the CNP, which could be attributed to degradation the graphene-metal interface as mentioned before.

In addition, for reproducing the measurements of the P-R configuration, the current leakage through contact S needs to be included. We notice in the experimental data (see Fig S16, Fig. S17 and Fig. S18) that the relative portions of the current flowing through contact S is smaller at higher doping where the graphene has lower resistance. Therefore, in a simple consideration, we assume a fixed conductivity for the leakage path σ_{leak}, which can be estimated by matching the proportion of the current branching out through contact S relative to the current continuing in the graphene ring.

Combining all together, we managed to find suitable simulation parameters (Table S2) that lead to simulated current distributions that align well with measurements at different experimental conditions overall (Fig. 2, Fig S13 and Fig. S14). We also verified that the simulated two-terminal resistance of the device is mostly determined by the graphene while the conductivities of other parts have little influence. Finally, the two-terminal contact resistance is taken into account as a constant offset added to the simulated two-terminal resistances. For Q-V and P-R contact configuration, they are estimated to be $R_{\mathrm{C}}^{QV} = 7.5\ \mathrm{k\Omega}$ and $R_{\mathrm{C}}^{PR} = 2.2\ \mathrm{k\Omega}$ to reach a good agreement between simulation and measurements (Fig. 1e and Fig. 4b).

Necessity of including interface resistances

We demonstrate the necessity of including non-uniform interface resistances in our hybrid device model by comparing the experimental data with simulations of different geometry models, as shown in Fig. S15.

We first look at current density maps ($a_1 - a_7$). As can be seen from a_2 and a_3, if the interface resistances are not included in the model, due to higher conductivity of the metal relative to that of the graphene around charge neutrality, the current would flow through the metal disc without being constrained to the graphene ring in the upper-left region and the lateral shift of the metal disc has negligible influence on the current distribution. In both cases, the current flow is symmetric about source and drain in the 180° contact configuration, which clearly contradicts with the measurements (a_1). Therefore, to explain our observation, non-uniform resistances breaking the spatial symmetry must be considered (a_4). The same conclusion holds in the hydrodynamic transport regime as shown in $a_5 - a_7$.

To reflect the interfacial properties of our hybrid device more straightforwardly, we resort to the current vorticity $\partial_x J_y - \partial_y J_x$. Without loss of generality, we explain in the framework of the Ohm's Law $-\sigma \boldsymbol{\nabla} \Phi = \mathbf{J}$ where all variables are assumed to be smooth over the space (in the case of boundary, the step function can be approximated by a smooth function, e.g. through a low pass spatial filter). By taking the spatial derivative of the Ohm's Law, we can obtain:

$$\begin{cases} -(\partial_y \sigma)(\partial_x \Phi) - \sigma \partial_{xy} \Phi = \partial_y J_x \\ -(\partial_x \sigma)(\partial_y \Phi) - \sigma \partial_{yx} \Phi = \partial_x J_y \end{cases} \tag{S20}$$

Using $\partial_{xy} \Phi = \partial_{yx} \Phi$ and $-\sigma \partial_{x(y)} \Phi = J_{x(y)}$, we get:

$$J_x \partial_y \sigma - J_y \partial_x \sigma = \sigma(\partial_x J_y - \partial_y J_x) \tag{S21}$$

which tells that generally at material interfaces where $\sigma(\mathbf{r})$ is non-uniform ($\boldsymbol{\nabla}\sigma \neq \mathbf{0}$), the current vorticity would be non-zero (provided that $\mathbf{J} \neq \mathbf{0}$).

Fig. S15b shows the current vorticity maps obtained from both measurements and simulations. It can be seen in b_2 that the finite vorticity appears at material boundaries (vacuum-graphene and graphene-metal) and is absent in the bulk of the material where $\sigma(\mathbf{r})$ is constant. Comparing b_3 with b_2, we see that the shift of the metal disc is manifested by a shift of the current vorticity pattern at circular graphene-metal boundary. In both cases, the patterns in the 180° contact configuration are symmetric about source and

drain. In contrast, the current vorticity of the measurement data (b_1) is asymmetric in the 180° contact configuration and exhibits a clear sign change at the upper-left graphene-metal boundary, signifying that in that region there exists an additional resistance layer with a lower conductivity compared to graphene. By including the non-uniform interface resistances in the model, the observed sign change can be reproduced (b_4). In the hydrodynamic regime ($b_5 - b_7$), due to the contribution from the $D_\nu \boldsymbol{\nabla}^2 \mathbf{J}$ term, the finite current vorticity is not only present at the material boundary but also spreads into the bulk of graphene, while its sign change at the upper-left graphene-metal boundary resulting from the non-uniform interface resistances is qualitatively the same.

3. SUPPLEMENTARY TABLES

Geometry parameters	Values		Obtained from:
Graphene channel width (W, w)	$(0.5\ \mu\mathrm{m}, 0.3\ \mu\mathrm{m})$		Design[a]
Graphene disc radius R_{out}	$1.2\ \mu\mathrm{m}$		Design[a]
Metal disc radius R_{in}	$0.9\ \mu\mathrm{m}$		Design[a]
Relative center shift Δx	$0.1\ \mu\mathrm{m}$		AFM
Graphene-metal interface width t	20 nm		see [b]
Angles specifying high/low-R_{C} sub-regions (α, β)	s_0	s_1 and s_2	Current maps
	$(43°, 152°)$	$(-20°, 250°)$	

Table S1. Geometry input parameters for the COMSOL simulations. $\mathrm{s}_{0,1,2}$ reflect different states of the device as the arc of high contact resistance increased during the study. [a]confirmed by AFM. [b] small enough number (limited by the mesh size) compared to other length scales for modeling the interface with finite contact resistance.

Materials	Model parameters	Values		Obtained from:
Graphene	Carrier density $n_{\mathrm{e/h}}$	$n_{\mathrm{e/h}}(k_{\mathrm{B}}T, E_{\mathrm{F}})$ (Eqn. S9)		Input parameters
	Zero-field mobility μ_0	1.4 $\mathrm{m}^2/\mathrm{V/s}$		see Methods
	Magnetotransport mobility μ_B	μ_0		same as μ_0 [a]
	Vorticity diffusion length D_ν	0.001 μm		see [b]
	Hall diffusion length D_H	0.001 μm		see [b]
Metal disc	Conductivity σ_{M}	8×10^{-4} S		Current maps
High R_c region	Contact resistance R_{C1}	$2 \times 10^3\ \Omega \cdot \mathrm{m}$		Current maps
Low R_c region	Contact resistance R_{C2}	s_0 and s_1	s_2	
		$4 \times 10^{-5}\ \Omega \cdot \mathrm{m}$	$2.5 \times 10^{-3}\ \Omega \cdot \mathrm{m}$	Current maps
Leakage at contact S	Conductivity σ_{leak}	s_0	s_1 and s_2	
		0	1.2×10^{-3} S	Current maps

Table S2. Main input material parameters for COMSOL simulation. $\mathrm{s}_{0,1,2}$ reflect different states of the device as the arc of high contact resistance increased during the study. [a] assuming weak electron-hole scattering. [b] close-to-zero value.

4. SUPPLEMENTARY FIGURES

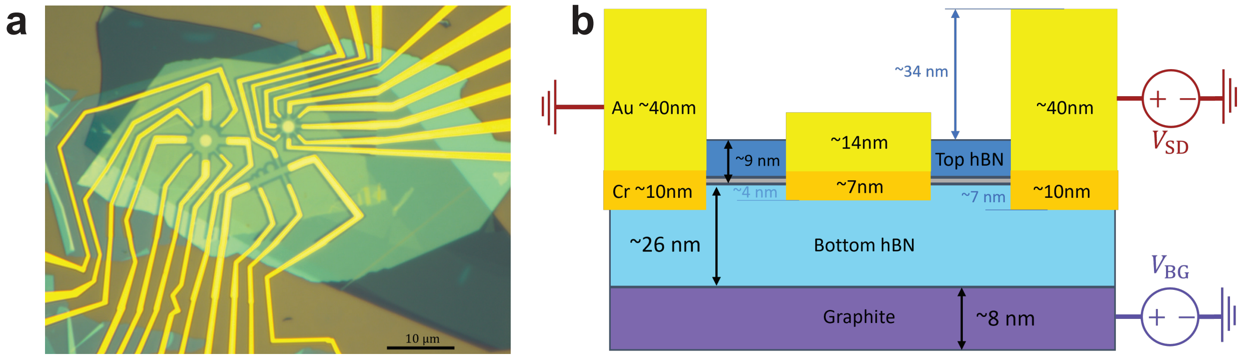

Figure S1. **Optical microscope image and the cross section schematic of the device.** **a,** Optical microscope image of the hBN-graphene-hBN-graphite stack and Au contacts. In total, three devices were fabricated on the same stack. All reported measurements are from the upper right device. The metal contact for graphite back-gate is at the bottom right outside the scope of the image. **b,** A schematic of the cross section of the measured device with the thickness of each layer and electrical connection scheme labeled. V_{SD} is the source-drain voltage and V_{BG} is the back-gate voltage.

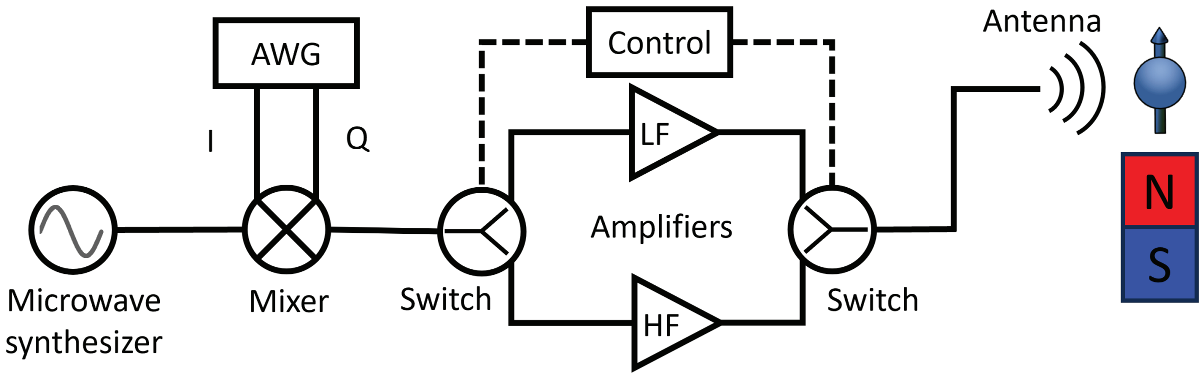

Figure S2. **Microwave circuit of the scanning NV magnetometer.** See the Methods section for more details. AWG = arbitrary waveform generator, LF = low frequency power amplifier, HF = high frequency power amplifier.

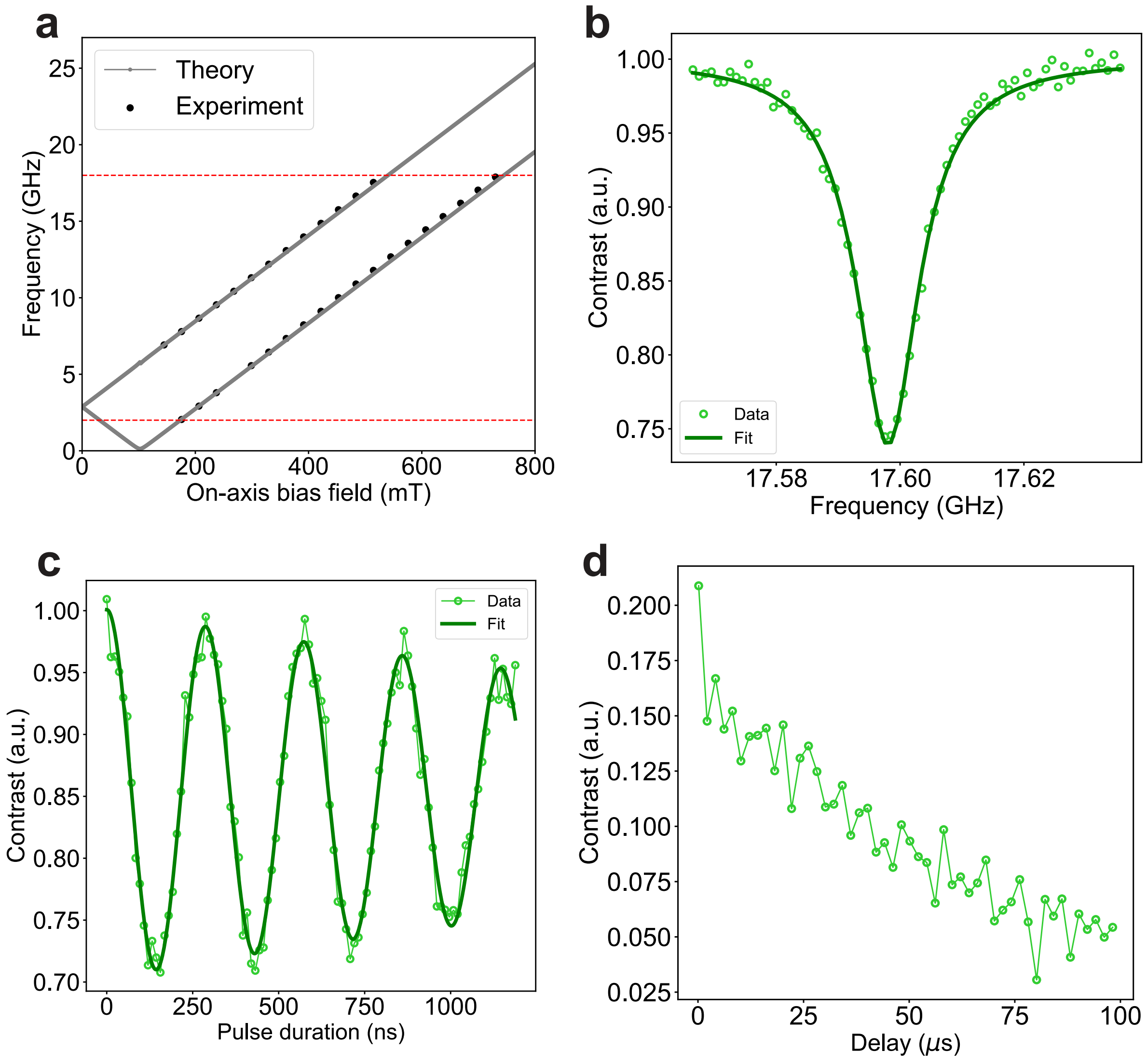

Figure S3. **Characterization of the NV center in the (111) diamond probe. a,** Plot of the spin resonance frequencies of the NV center as a function of on-axis bias magnetic field. Grey lines correspond to theory predictions based on the Zeeman term in the NV Hamiltonian and black dots correspond to the extracted resonance frequency from ODMR measurements. The red dashed lines mark the frequency range supported by the RF circuit. **b-d,** Sensor characterization at around 0.53 T, roughly the highest bias magnetic field at which the scanning was conducted in this work. **b,** ODMR measurement. The resonance is at $f_{\text{res}} \approx 17.598$ GHz with a contrast of approximately 26%. **c,** Rabi measurement: The Rabi frequency is $f_{\text{rabi}} \approx 3.5$ MHz. **d,** Echo measurement: The coherence time is $T_2^{\text{echo}} > 25\ \mu$s.

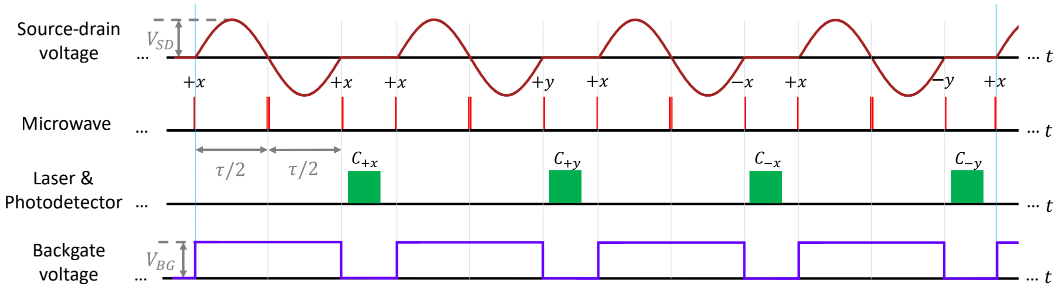

Figure S4. **Timing diagram of the four-phase echo measurement protocol used for AC current imaging.** τ is the total evolution time of one echo sequence. $\pm x$ and $\pm y$ indicate pulse phases, and $C_{\pm x}$ and $C_{\pm y}$ the respective photon counts, as explained in Methods.

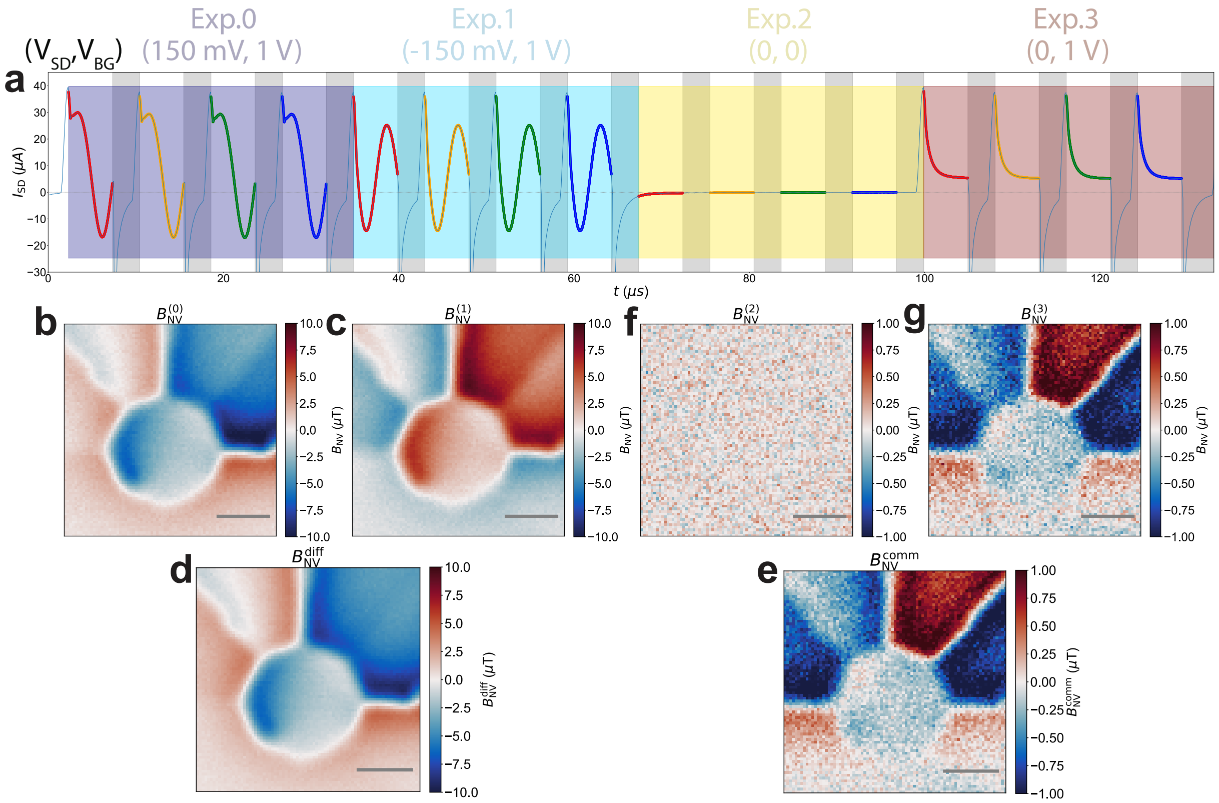

Figure S5. **Differential current imaging technique used for separating source-drain and back-gate-leakage currents.** The data shown was obtained with P-R as source-drain contacts at low bias field strength < 10 mT. **a,** Recorded traces of the current flowing through the device during one measurement cycle containing 4 sets of experiments (Exp. 0-3). **b,(c,)** B_{NV} images of Exp. 0(1), measured with $V_{\mathrm{SD}} = 150(-150)\,\mathrm{mV}$ and $V_{\mathrm{BG}} = 1\,\mathrm{V}$. Both images show B_{NV} signals along contacts besides P and R, indicating current leakage. **d,** Differential-mode image: $B_{\mathrm{NV}}^{\mathrm{diff}} = (B_{\mathrm{NV}}^{(0)} - B_{\mathrm{NV}}^{(1)})/2$ originating from the current induced by V_{SD}, which shows that after the current is injected from the source P, it flows through both contacts R and S, indicating a potential short circuit between the two. **e,** Common-mode image: $B_{\mathrm{NV}}^{\mathrm{comm}} = (B_{\mathrm{NV}}^{(0)} + B_{\mathrm{NV}}^{(1)})/2$ originating from the current induced by V_{BG} only, which shows that the leakage current flows through multiple contacts from P to T. Its strength is at least $5\times$ weaker than the source-drain current according to its peak-to-peak value compared to **b(c)**. **f,** B_{NV} image of Exp. 2($V_{\mathrm{SD}} = 0$ and $V_{\mathrm{BG}} = 0$) as a reference measurement. As expected, no clear pattern appears. **g,** B_{NV} image of Exp. 3($V_{\mathrm{SD}} = 0$ and $V_{\mathrm{BG}} = 1\,\mathrm{V}$), which shows similar features with close peak-to-peak values compared to **e**. This is contributed by the back-gate-leakage current as can be seen from the recorded current traces in **a**, verifying that contacts P to T are not perfectly isolated from the back-gate. Scale bars, $1\,\mu$m.

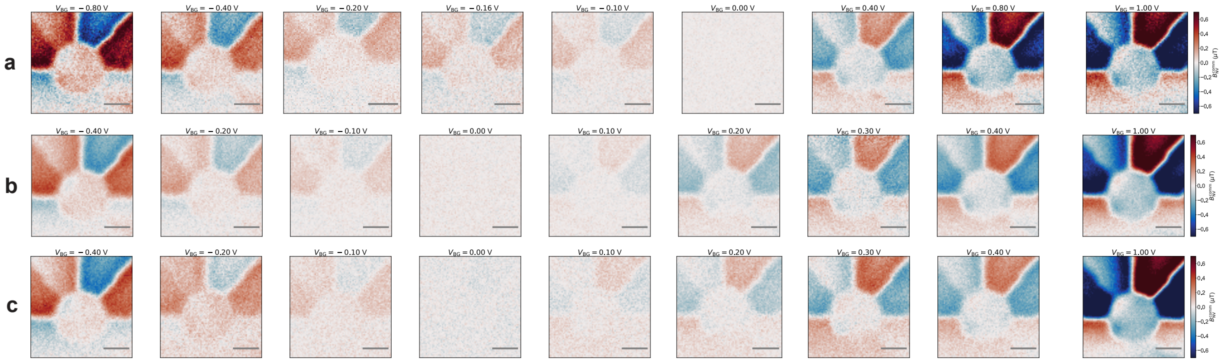

Figure S6. **Back-gate voltage dependence of $B_{\mathrm{NV}}^{\mathrm{comm}}$ indicating back-gate-leakage currents. a,** Measurements at 0.54 T. **b,** Measurements at 0.25 T. **c,** Measurements at 0.04 T. All images are obtained in the same way as Fig. S5e. Consistent across the 3 sets of measurements, no signatures of leakage currents are present at $V_{\mathrm{BG}} = 0$, while the pattern becomes more prominent as $|V_{\mathrm{BG}}|$ increases and is of opposite sign for positive and negative V_{BG}. Scale bars, $1\,\mu$m.

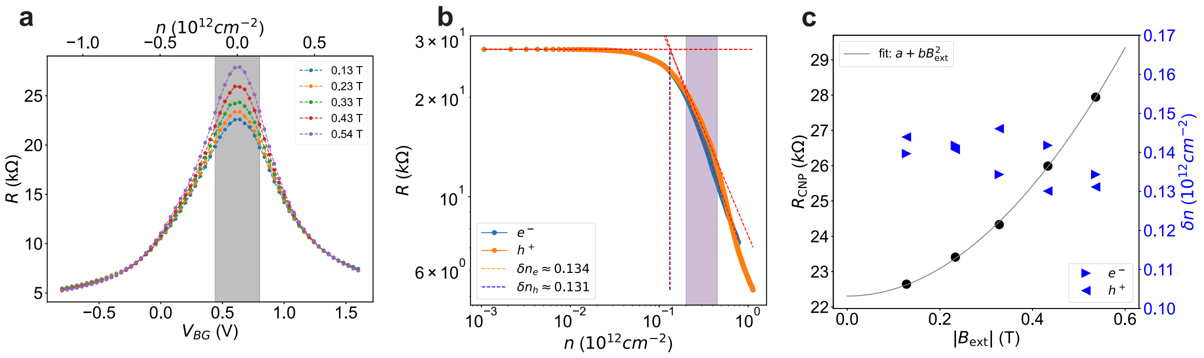

Figure S7. **Bulk resistance measurements (Part 1).** **a,** Conventional characterization of the two-terminal resistance (with P-R contact configuration) as a function of back-gate voltage $V_{\rm BG}$ under different bias magnetic field strength $|B_{\rm ext}|$. The resistance maximum (at the CNP) is reached at $V_{\rm BG} \approx 0.62$ V. The deviation from zero potentially originates from the presence of residual charges in the device. The gray region marks the regime where $|E_{\rm F}| \leq k_{\rm B}T$, corresponding to a carrier density $|n| \leq 0.144 \times 10^{12}$ cm^{-2}. It can be seen that the resistance R exhibits a more prominent increase with $|B_{\rm ext}|$ in this regime compared to the outside (non-gray) region where R stays almost constant at different $|B_{\rm ext}|$. **b,** Data from **a** at the highest bias field strength (0.54 T) replotted on a logarithmic scale. Dashed lines are linear fits to the data in the low- and high density regimes, respectively. From the fits, the charge density fluctuations δn are extracted [1]. Resulting values are $\delta n \sim 0.134 \times 10^{12}$ cm^{-2} for electrons and $\delta n \sim 0.131 \times 10^{12}$ cm^{-2} for holes. **c,** Extracted resistance maximum $R_{\rm CNP}$ (black dots with the left axis) and charge density fluctuation $\delta n_{\rm e/h}$ (blue triangles with the right axis) at different $|B_{\rm ext}|$. The former can be fitted using a parabolic function $a + bB^2_{\rm ext}$ (with $a = 22.31 \pm 0.04$ and $b = 19.54 \pm 0.25$) shown as the gray curve, which aligns with the intrinsic MR effect in charge-neutral MLG [1]. The latter shows that δn is consistently around 0.14×10^{12} cm^{-2} for both electrons and holes in our device.

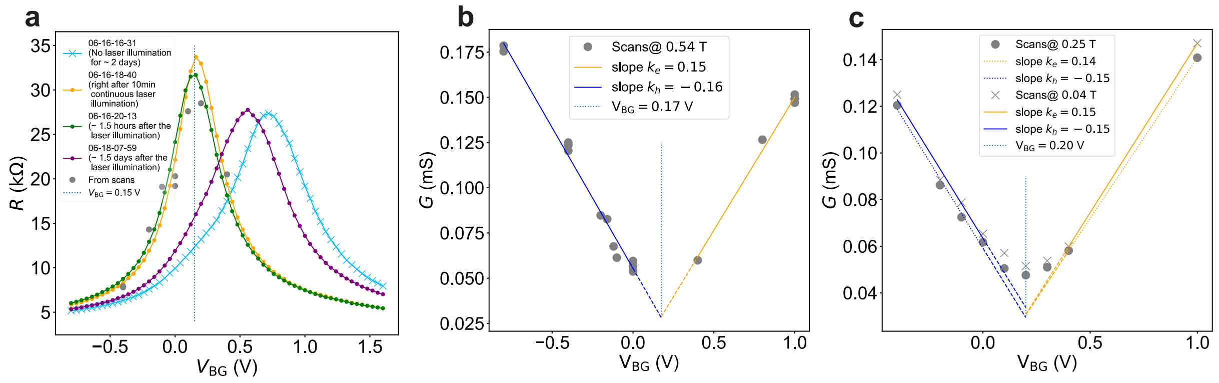

Figure S8. **Bulk resistance measurements (Part 2).** **a,** Two-terminal resistance as a function of back-gate voltage V_{BG} measured at the highest bias magnetic field strength ($B_{\mathrm{ext}} = 0.54$ T) under different conditions: no laser illumination for ~ 2 days (blue), right after continuous laser illumination for ~ 10 minutes (orange), ~ 1.5 hours after the laser illumination (green), ~ 1.5 days after the laser illumination (purple). It can be seen that while the CNP of the device was at $V_{\mathrm{BG}} \approx 0.62$ V before laser illumination, it shifted towards smaller V_{BG} once the device was illuminated, and settled around $V_{\mathrm{BG}} \approx 0.175$ V for times longer than ca. 10 min. In addition, an enhancement of the resistance around the CNP was observed, which can be interesting for future investigation. In the absence of laser illumination, the CNP gradually relaxed back to the initial value albeit on a much longer timescale compared to the photodoping process, consistent with previous studies [7, 8]. Gray dots correspond to resistances extracted from the magnetometry scans under the same bias magnetic field and align well with the resistance curve measured upon laser illumination. This is to be expected because the device is subject to laser pulses during the quantum phase measurement (Fig. S4). **b,** Conductance $G = 1/R$ at 0.54 T as a function of V_{BG} and the corresponding linear fit to the single-carrier model. The fitted slope (that is proportional to the mobility μ) exhibits similar absolute value for electron-doping (0.15) and hole-doping (0.16), justifying the assumption of $\mu_{\mathrm{e}} \approx \mu_{\mathrm{h}}$ in our device. The two lines intersect at around 0.17 V, which agrees well with the CNP estimation from transport curves measured upon laser illumination in **a**. **c,** Same as **b** for lower bias fields (0.25 T, 0.04 T). The fitted results support the previous claims.

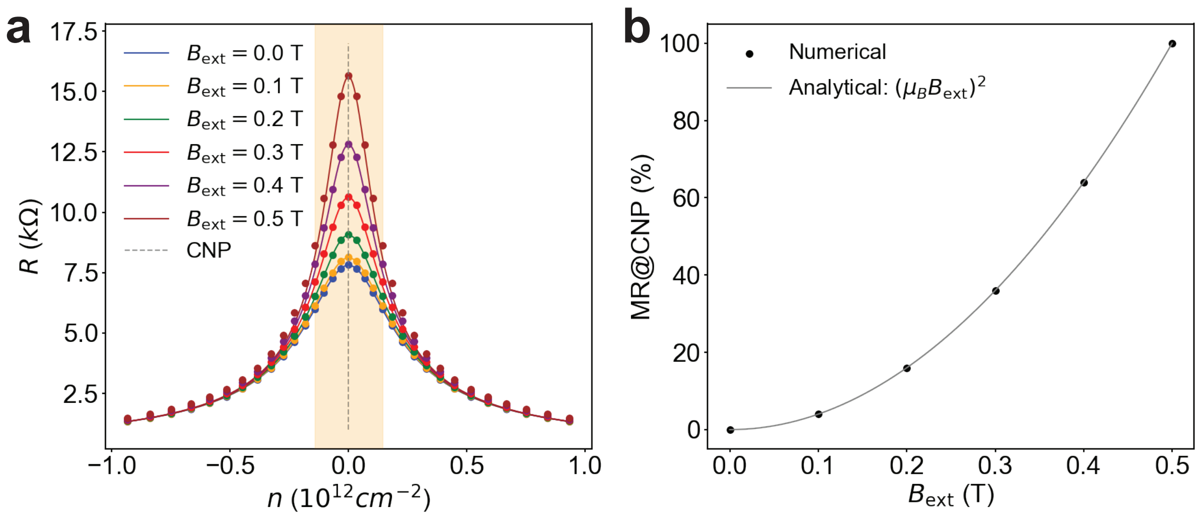

Figure S9. **Verification of the two-carrier (diffusive) model in a channel geometry (simulation). a,** Two-terminal resistance R as a function of (net) carrier density n at different out-of-plane bias fields $B_{\rm ext}$. Curves and dots correspond to the analytical and numerical (COMSOL) results, respectively. **b,** MR at CNP($n = 0$) as a function of $B_{\rm ext}$.

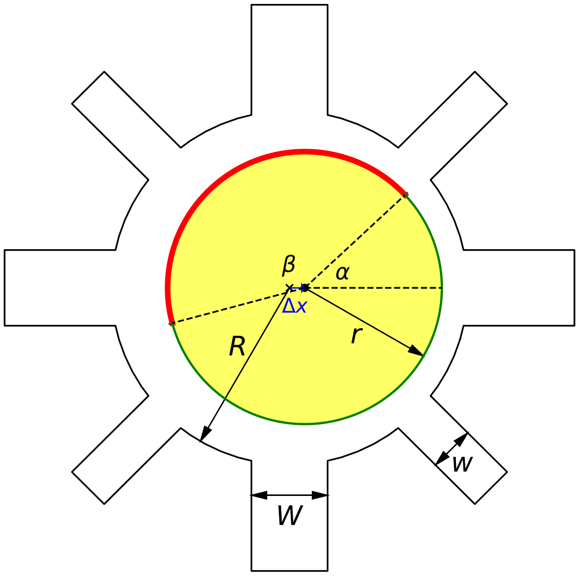

Figure S10. **Device model**. The white and yellow area inside the device boundary (black) correspond to graphene and metal regions, respectively. Thick red and thin green contours indicate sections of high and low contact resistance at the graphene-metal interfaces, respectively. The values of the geometry parameters marked in the schematic are listed in Table S1.

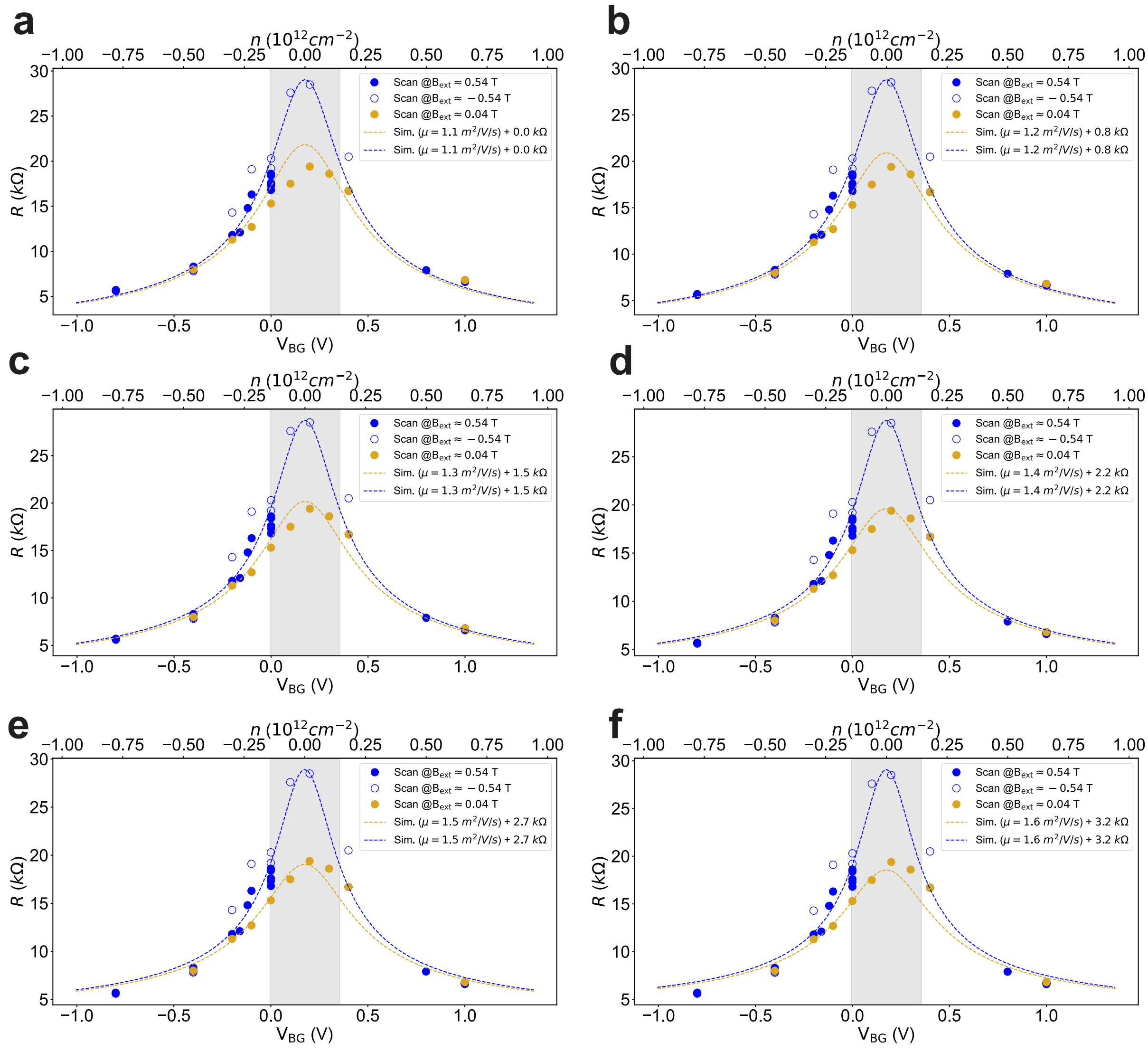

Figure S11. **Estimation of carrier mobility from two-terminal resistances**. **a-f,** Comparison of carrier-density-dependent two-terminal resistances between experiments (dots) and simulations (curves) with different carrier mobilities μ (from 1.1 $m^2/V/s$ to 1.6 $m^2/V/s$). For each μ, the two-terminal contact resistance R_C^{PR} is determined by minimizing the total difference between measurement data and the corresponding simulated values. The best estimate for the mobility is $\mu = 1.35 \pm 0.25\,\mathrm{m^2/V/s}$.

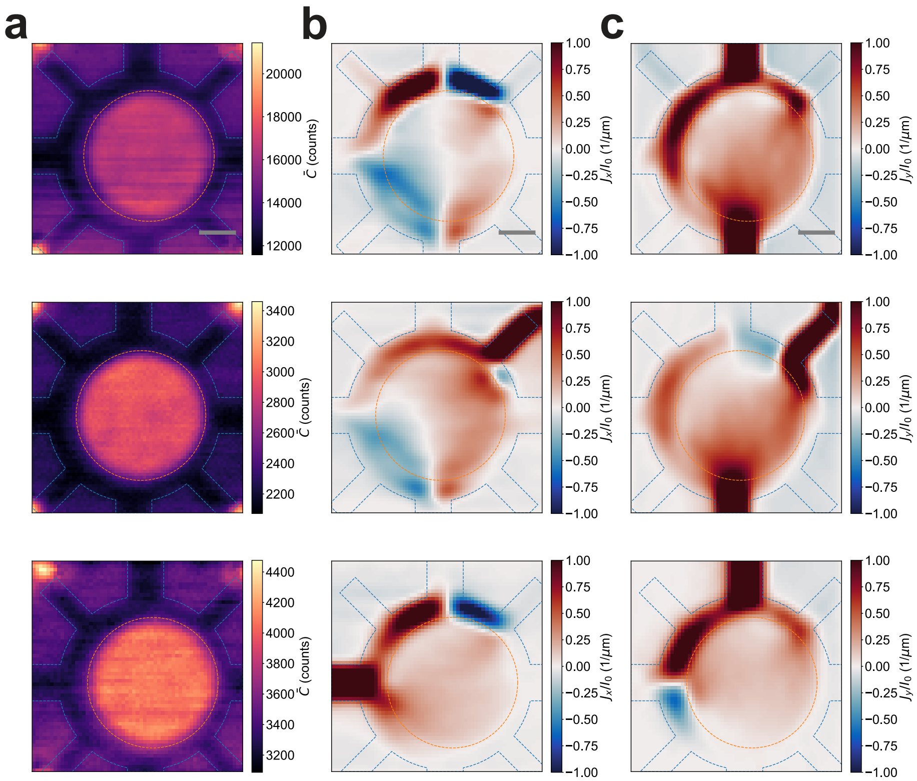

Figure S12. **Supplementary data for Fig. 2 in the main text.** **a,** NV PL maps used for aligning the device geometry to the magnetometry scans. **b**(**c**), Reconstructed $J_x(J_y)$ images from the experimental data. Scale bars, $0.5\,\mu$m.

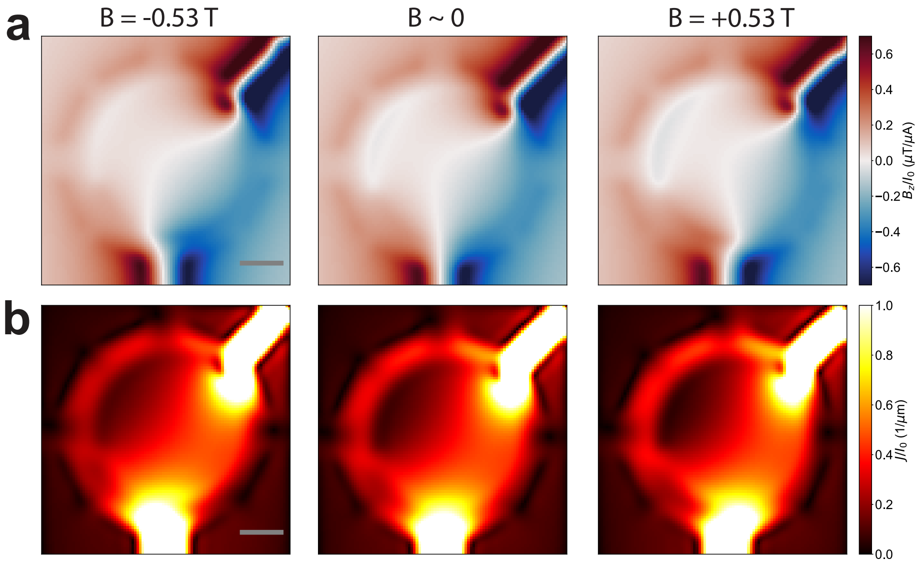

Figure S13. **Supplementary simulation for Figs. 3a,b in the main text.** Simulated magnetic field B_z (**a**) and current density J (**b**) maps for direct comparison with the experimental data shown in Fig. 3a(b). The simulations used model s_0. Scale bars, 0.5 μm.

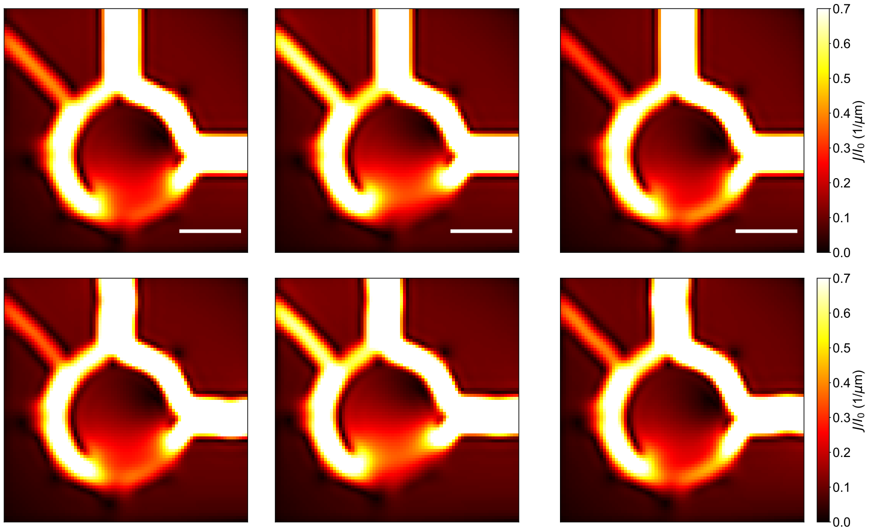

Figure S14. **Supplementary simulation for Fig. 4d in the main text.** Simulated current density maps for direct comparison with the experimental data shown in Fig. 4d. The simulations used model s_1. Scale bars, 1 μm.

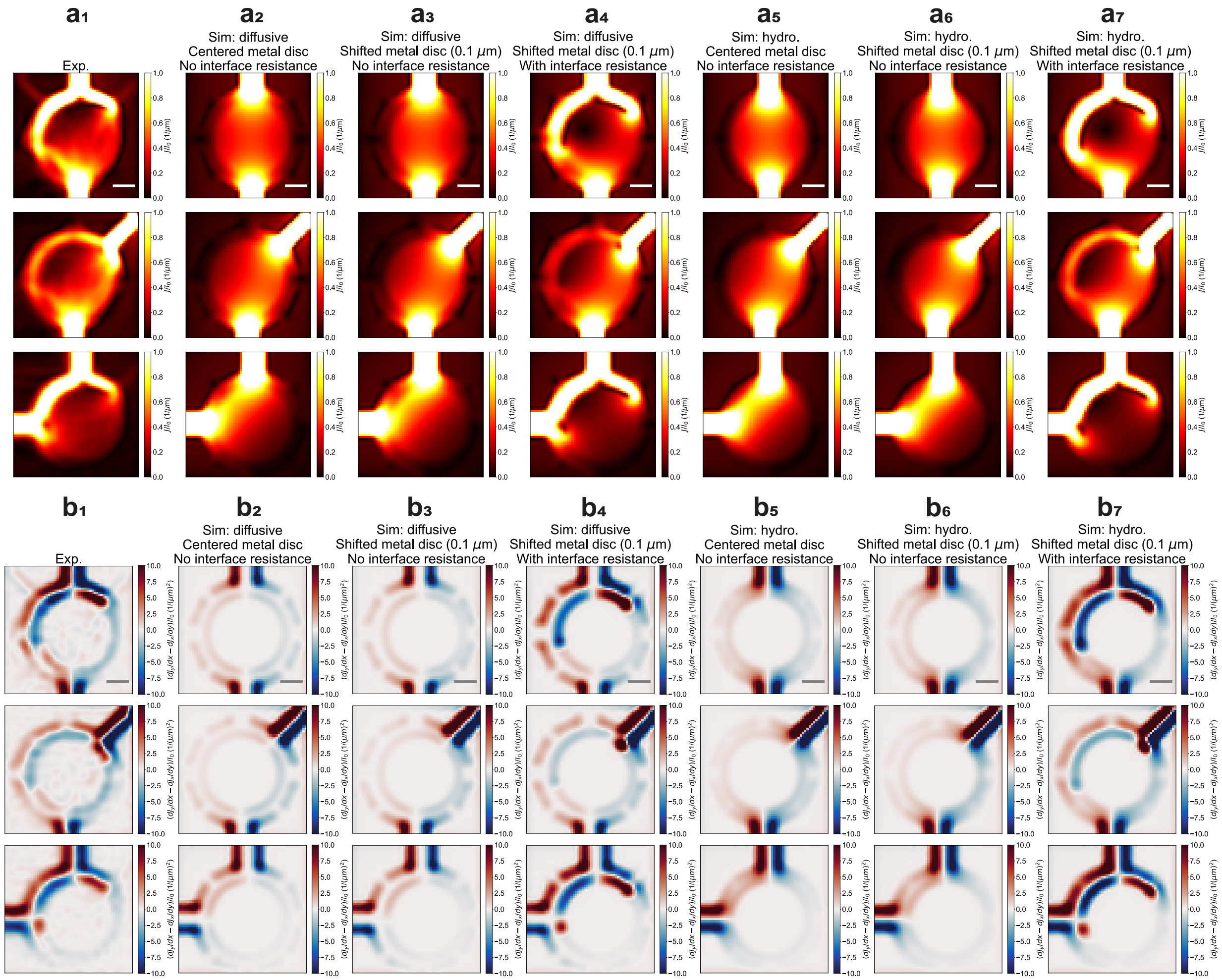

Figure S15. **Supplementary data for demonstrating the existence of non-uniform interface resistances along the circular graphene-metal boundary.** (**a**) Current density maps. (**b**) Current vorticity maps. Column 1 corresponds to the measurements. where $\mathbf{a}_1$ is the same as Fig. 2c in the main text. Column 2-4 and 5-7 present the diffusive ($D_\nu = 0.001$ μm) and hydrodynamic ($D_\nu = 0.1$ μm) simulations using different geometry models of the hybrid device: Column 2 and 5 corresponds to centered metal disc *without* graphene-metal interface resistance; Column 3 and 6 corresponds to shifted metal disc ($\Delta x = 0.1$ μm) *without* graphene-metal interface resistance; Column 4 and 7 corresponds to shifted metal disc ($\Delta x = 0.1$ μm) *with* graphene-metal interface resistance, where $\mathbf{a}_4$ is the same as Fig. 2d in the main text. Scale bars, 0.5 μm.

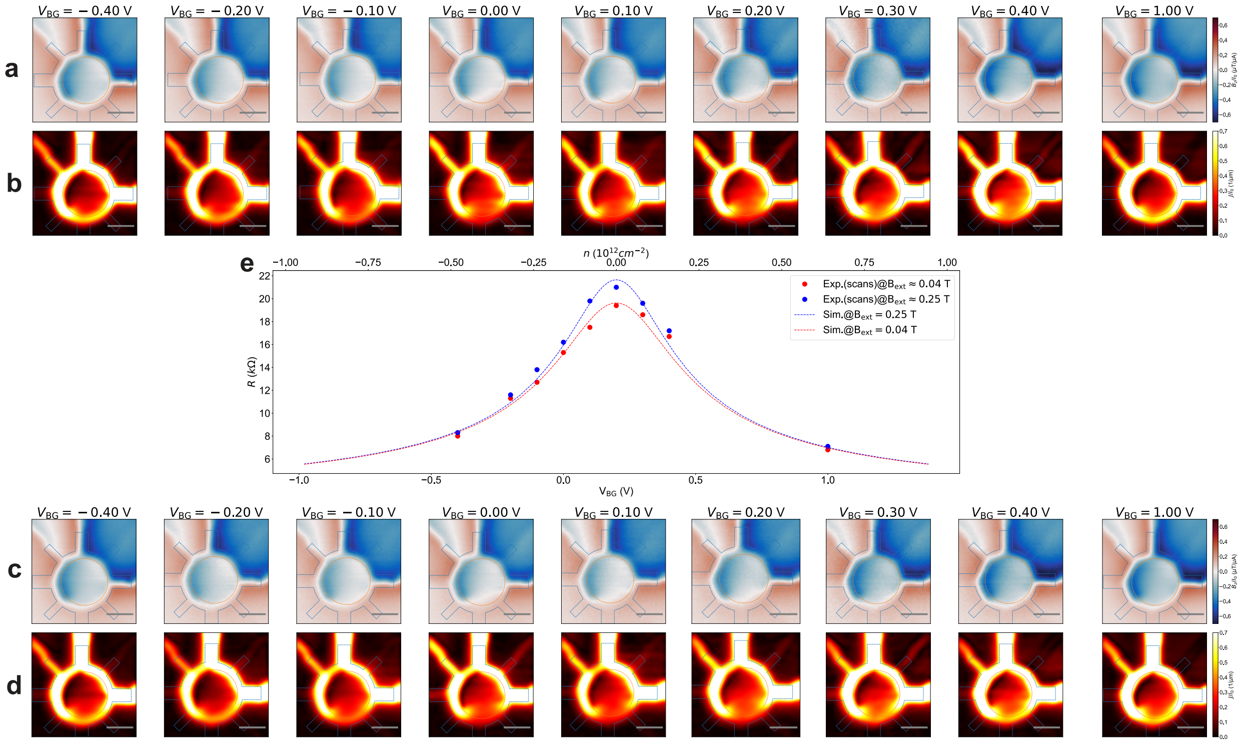

Figure S16. **Supplementary measurement data: carrier doping dependence at bias magnetic fields** $0.25\,\mathrm{T}$ **and** $0.04\,\mathrm{T}$**. a,(c,)** Measured out-of-plane stray magnetic field B_z at different back-gate voltages V_{BG} under $0.25(0.04)\,\mathrm{T}$. **b,(d,)** The reconstructed current density J under $0.25(0.04)\,\mathrm{T}$, which aligns with device model s_1. Scale bars, $1\,\mu$m. **e,** Two-terminal resistance as a function of V_{BG} (carrier density n). The dots correspond to the resistance extracted from the transport data recorded simultaneously with the magnetometry scans. The dashed curve shows the corresponding two-carrier-model fit.

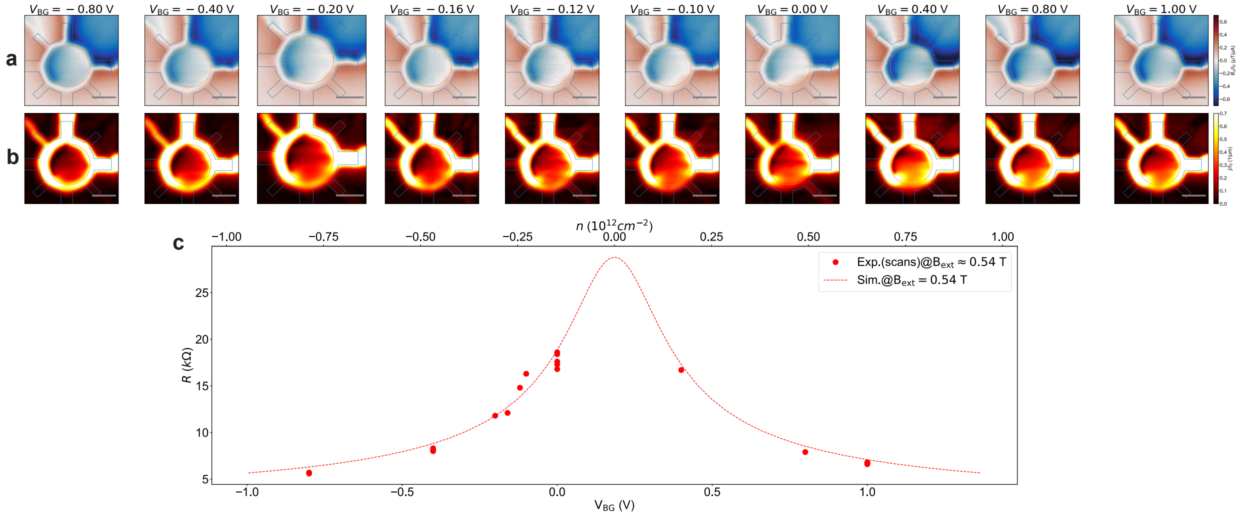

Figure S17. **Supplementary measurement data: carrier doping dependence at bias magnetic field** $0.54\,\mathrm{T}$**. a,** Measured out-of-plane stray magnetic field B_z at different back-gate voltages V_{BG}. **b,** The reconstructed current density J, which aligns with device model s_1. Scale bars, $1\,\mu$m. **c,** Two-terminal resistance as a function of V_{BG} (carrier density n). The dots correspond to the resistance extracted from the transport data recorded simultaneously with the magnetometry scans. The dashed curve shows the corresponding two-carrier-model fit.

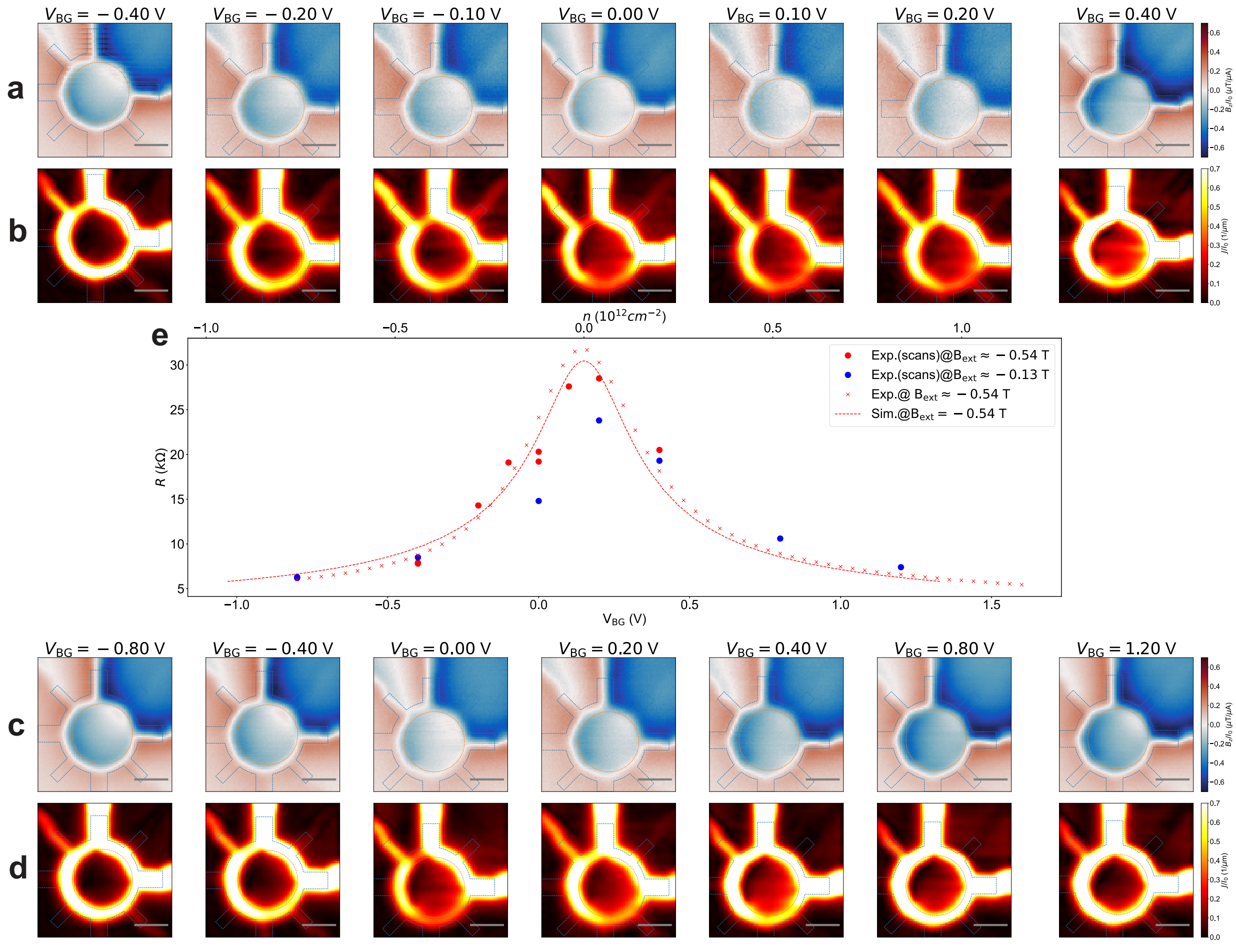

Figure S18. **Supplementary measurement data: carrier doping dependence at bias magnetic fields** $-0.54\,\mathrm{T}$ **and** $-0.13\,\mathrm{T}$**. a,(c,)** Measured out-of-plane stray magnetic field B_z at different back-gate voltages V_{BG} under $-0.54(-0.13)\,\mathrm{T}$. **b,(d,)** The reconstructed current density J under $-0.54(-0.13)\,\mathrm{T}$, which aligns with device model s_2. Scale bars, $1\,\mu\mathrm{m}$. **e,** Two-terminal resistance as a function of V_{BG} (carrier density n). The dots correspond to the resistance extracted from the transport data recorded simultaneously with the magnetometry scans. The crosses show the transport data as a function of back-gate voltage V_{BG} at $-0.54\,\mathrm{T}$. The dashed curve shows the corresponding two-carrier-model fit.

SUPPLEMENTARY REFERENCES

---

[1] N. Xin, J. Lourembam, P. Kumaravadivel, A. Kazantsev, Z. Wu, C. Mullan, J. Barrier, A. A. Geim, I. Grigorieva, A. Mishchenko, *et al.*, Giant magnetoresistance of dirac plasma in high-mobility graphene, Nature **616**, 270 (2023).

[2] J. Martin, N. Akerman, G. Ulbricht, T. Lohmann, J. v. Smet, K. Von Klitzing, and A. Yacoby, Observation of electron–hole puddles in graphene using a scanning single-electron transistor, Nature physics **4**, 144 (2008).

[3] A. Aharon-Steinberg, T. Völkl, A. Kaplan, A. K. Pariari, I. Roy, T. Holder, Y. Wolf, A. Y. Meltzer, Y. Myasoedov, M. E. Huber, *et al.*, Direct observation of vortices in an electron fluid, Nature **607**, 74 (2022).

[4] M. L. Palm, C. Ding, W. S. Huxter, T. Taniguchi, K. Watanabe, and C. L. Degen, Observation of current whirlpools in graphene at room temperature, Science **384**, 465 (2024).

[5] A. I. Berdyugin, S. G. Xu, F. M. D. Pellegrino, R. Krishna Kumar, A. Principi, I. Torre, M. Ben Shalom, T. Taniguchi, K. Watanabe, I. V. Grigorieva, M. Polini, A. K. Geim, and D. A. Bandurin, Measuring Hall viscosity of graphene's electron fluid, Science **364**, 162 (2019).

[6] T. H. Hewett and F. V. Kusmartsev, Extraordinary magnetoresistance: sensing the future, Central European Journal of Physics **10**, 602 (2012).

[7] L. Ju, J. Velasco, E. Huang, S. Kahn, C. Nosiglia, H. Z. Tsai, W. Yang, T. Taniguchi, K. Watanabe, Y. Zhang, G. Zhang, M. Crommie, A. Zettl, and F. Wang, Photoinduced doping in heterostructures of graphene and boron nitride, Nature Nanotechnology **9**, 348 (2014).

[8] M. L. Palm, W. S. Huxter, P. Welter, S. Ernst, P. J. Scheidegger, S. Diesch, K. Chang, P. Rickhaus, T. Taniguchi, K. Watanabe, K. Ensslin, and C. L. Degen, Imaging of submicroampere currents in bilayer graphene using a scanning diamond magnetometer, Physical Review Applied **17**, 10.1103/physrevapplied.17.054008 (2022).
