# Supplementary material for: Direct imaging of magnetotransport at graphene-metal interfaces with a single-spin quantum sensor
Source: Nat Commun. 2026 May 28;17:6913. doi: 10.1038/s41467-026-73480-8 (PMC13389063; doi:10.1038/s41467-026-73480-8)
Supplement: Supplementary file 2 — Peer Review File [file 41467_2026_73480_MOESM2_ESM.pdf]

nature portfolio

Peer Review File

Direct imaging of magnetotransport at graphene-metal interfaces with a single-spin quantum sensor

Corresponding Author: Professor Christian Degen

This file contains all reviewer reports in order by version, followed by all author rebuttals in order by version.

Version 0:

Reviewer comments:

Reviewer #1

(Remarks to the Author)
Comments
In this manuscript, C. Ding et al. report nanoscale imaging of magnetotransport in a graphene–metal hybrid device using scanning magnetometry based on a diamond NV center. Although magnetoresistance has been extensively studied, particularly through current-transport measurements, this study extends prior work by simultaneously imaging local current profiles while performing transport measurements. The authors find good agreement between the imaging results and the transport data, and they also observe interesting features that cannot be easily inferred from transport measurements alone. These features are interpreted as arising from a combination of intrinsic magnetoresistance near the Dirac cone, carrier hydrodynamics, and contact resistances between graphene and the metal electrodes. The results provide valuable insight into the potential of nanoscale imaging methods for investigating a wide range of transport phenomena.
Based on the results and the well-analyzed, well-written manuscript, I recommend publication in Nature Communications after the authors address the following questions.

Major questions and suggestions:
(1) Concerning the interface resistance
1. The center of the circular metal disk appears to be displaced by ~ 0.1 μm, causing the graphene channel at the upper-left region to be roughly 160% wider than that at the lower-right region. Could this geometric asymmetry alone account for the observed difference in current density (e.g. ~ 200%)? Is the inclusion of interface resistance still necessary to interpret the data?
2. Following Question #1, it is unclear whether the authors used a diffusive or a hydrodynamic transport regime in the finite element simulations employed to extract the interface resistance. The modeling seems to assume diffusive behavior, while later the manuscript argues that zero bias and zero magnetic field correspond to a hydrodynamic regime. This discrepancy may significantly affect the extracted interface resistance values. The authors are encouraged to clarify which regime was assumed and justify its validity.
3. Continuing from Question #2, in Fig. 4a, the authors attribute the enhanced current in the upper-left graphene channel to hydrodynamic flow at zero magnetic field, transitioning to a diffusive regime at higher fields (e.g., Fig. 3b). However, in Fig. 4d, the current profiles in the lower-right region at zero bias (VBG = 0 V) exhibit no clear difference between zero and high magnetic fields. What causes this discrepancy between the two regions? Does this suggest a significant role of the interface resistance? If so, by what mechanism does the interface resistance lead to such distinct current profiles in the hydrodynamic versus diffusive regimes?
If the interface resistance suppresses current transfer from graphene into the metal disk in the hydrodynamic regime, then why does such suppression appear to occur at the upper-left region but not at the lower-right region? Is this discrepancy attributable to a difference in interface resistance between the two regions? If so, the authors should specify the approximate range of interface resistance at which it begins to alter the current profiles across the hydrodynamic–diffusive transport crossover, at least based on their simulations.
The authors also mention possible degradation of the graphene–metal interface over time in the supplementary materials, which could alter the interface resistance. Did the enhanced current in the upper-left region diminish over time as a result of a reduced interface resistance, or, conversely, did the current in the lower-right region increase due to an increased interface resistance?
Overall, a more careful comparison of the current profiles in the two regions around the disk—explicitly distinguishing hydrodynamic vs diffusive transport and clarifying the role of interface resistance in these regimes—would help strengthen

the authors' conclusions.

(2) Concerning the intrinsic magnetoresistance (MR)
In Fig. 3e, the graphene current (%) at zero fields suddenly drops at higher magnetic fields and then remains nearly constant beyond approximately ±0.1 T. On the other hand, the resistance measurements near CNP in Fig. 4b show a noticeable difference between zero field and ±0.54 T, which the authors interpret as evidence of intrinsic MR.
It would be helpful for the authors to provide resistance data at intermediate magnetic fields (e.g., 0.1 T to 0.5 T) and discuss whether the resistance remains nearly constant in this range, consistent with the current imaging data in Fig. 3e. This comparison would clarify whether the magnetoresistance originates from intrinsic transport properties or could instead be explained by a transition between hydrodynamic and diffusive regimes or by variations in interface resistance.
Moreover, the cited reference 24 reports a linear MR in this field regime. The present measurements (i.e., no field dependence near CNP based on Fig. 3e) seems to be inconsistent with the reference and wonder if they provide sufficient evidence to claim the intrinsic MR. If the intrinsic MR is indeed the origin, the authors are encouraged to suggest its potential origins more explicitly.

Minor corrections and suggestions:
1. Replace "change neutral point (CNP)" with "charge neutral point (CNP)".
2. Page 8, line 5: I suggest to modify the text to "…the Q–V contact configuration (135°)…" for improved clarity.

Reviewer #2

(Remarks to the Author)
The work of Ding et al. reported the high resolution direct imaging of magnetotransport by using a scanning diamond sensor under magnetic field up to 0.5 T. The results reveals the distribution of electrical current at nanoscale and the properties of a graphene-metal hybrid device. The work shows the potential of solid-state spins to study complex electrical phenomenon. There are several comments that should be addressed:

1, Permanent magnets were used this work to produce high magnetic field. How was the inhomogeneity of the magnetic distribution? Did it affect the spin manipulation and sensing in the area of interest? As it is important to make the bias magnetic vector parallel with NV center axis, it will be helpful to provide more details about the adjusting of magnetic field.

2, In addition, microwave was transmitted through an aluminum wire for NV center spin manipulation. Will the metallic electrodes of hybrid graphene-metal device change the local microwave distribution, and subsequently reduce the spin manipulation fidelity?

3, How was the sensitivity of NV center under high magnetic field?

4, The current with a frequency of 30 – 300 kHz was detected. Was the frequency kept the same in Fig.1-4? If not, please give the exact value. Will the transport properties of device change with current frequency?

5, In Fig 4d, small current leakage was also observed at contacts Q and W, which was absent in the simulation of Fig S14. And it changed with the magnetic field, while VBG = 0V. Can the author give a brief explanation?

Version 1:

Reviewer comments:

Reviewer #1

(Remarks to the Author)
Comments
The authors have carefully addressed all of my questions and have reflected them in the main text, figures, and supplementary materials. Therefore, I recommend publication of the manuscript, except for the following issues, which require clarification.

Authors' response to Reviewer #1:
"In response, we add Fig. S15 and Subsection 2.2 ("Necessity of including interface resistances") to the SI. In addition to the current density $|\boldsymbol{J}|$ [Fig. S15, a1-a7], we now also show the current vorticity ($\partial xJy - \partial yJx$) [Fig. S15, b1-b7], which clearly delineates to locations of high interface resistance."
Reviewer #1:
I cannot find the newly added Fig. S15; it appears to be identical to the previous version. I am also confused by the panel labeling (e.g., a1). It is possible that the updated figure did not appear correctly when the file was saved as a PDF. Please double-check this.

Authors' response to Reviewer #1:
"P7, Fig. 3e caption: We likewise adjusted the text to clarify that Fig. 1e and Fig. 3 were measured concurrently."
Reviewer #1:

I can find the corrected statement in the caption of Fig. 1e, but not in the caption of Fig. 3e or Fig. 3. Please double-check this as well.

Reviewer #2

(Remarks to the Author)
The authors have answered all my comments. I recommend the publication.

Open Access This Peer Review File is licensed under a Creative Commons Attribution 4.0 International License, which permits use, sharing, adaptation, distribution and reproduction in any medium or format, as long as you give appropriate credit to the original author(s) and the source, provide a link to the Creative Commons license, and indicate if changes were made.
In cases where reviewers are anonymous, credit should be given to 'Anonymous Referee' and the source.
The images or other third party material in this Peer Review File are included in the article's Creative Commons license, unless indicated otherwise in a credit line to the material. If material is not included in the article's Creative Commons license and your intended use is not permitted by statutory regulation or exceeds the permitted use, you will need to obtain permission directly from the copyright holder.
To view a copy of this license, visit https://creativecommons.org/licenses/by/4.0/

Reviewer #1 (Remarks to the Author):

In this manuscript, C. Ding et al. report nanoscale imaging of magnetotransport in a graphene–metal hybrid device using scanning magnetometry based on a diamond NV center. Although magnetoresistance has been extensively studied, particularly through current-transport measurements, this study extends prior work by simultaneously imaging local current profiles while performing transport measurements. The authors find good agreement between the imaging results and the transport data, and they also observe interesting features that cannot be easily inferred from transport measurements alone. These features are interpreted as arising from a combination of intrinsic magnetoresistance near the Dirac cone, carrier hydrodynamics, and contact resistances between graphene and the metal electrodes. The results provide valuable insight into the potential of nanoscale imaging methods for investigating a wide range of transport phenomena.
Based on the results and the well-analyzed, well-written manuscript, I recommend publication in Nature Communications after the authors address the following questions.

Reply: We thank the Reviewer for the detailed assessment and the positive evaluation of our work. We address the questions point-by-point below.

Major questions and suggestions:
(1) Concerning the interface resistance
1. The center of the circular metal disk appears to be displaced by ~ 0.1 μm, causing the graphene channel at the upper-left region to be roughly 160% wider than that at the lower-right region. Could this geometric asymmetry alone account for the observed difference in current density (e.g. ~ 200%)? Is the inclusion of interface resistance still necessary to interpret the data?

Reply: The geometric asymmetry alone cannot account for the observed difference in current density, and the inclusion of interface resistance is mandatory to explain our measurements. To clarify this, we carried out additional simulations for direct comparison with the experimental data in Fig. 2. These simulations cover different possible states/models of the hybrid device, including a centered metal disc without interface resistance, a shifted metal disc (0.1 μm) without interface resistance, and shifted metal disc (0.1 μm) with interface resistance. The simulations are shown in new Fig. S15.

As can be seen from panels **a2** and **a3**, when the interface resistances are not included in the model, the current flows through the metal disc without being constrained to the graphene ring in the upper-left region, regardless of whether the metal disc is centered or not. This is due to the higher conductivity of the metal relative to that of the graphene around charge neutrality. In addition, the lateral shift of metal disc does not break the source-drain symmetry in the 180° configuration, and therefore cannot account for the large source-drain asymmetry observed in the experimental current distribution. Only a non-uniform interface resistance along the circular graphene-metal boundary reproduces the asymmetry, as shown in **a4**. This conclusion also holds in the hydrodynamic transport regime as shown in (**a5**-**a7**).

In response, we add Fig. S15 and Subsection 2.2 (“Necessity of including interface resistances”) to the SI. In addition to the current density $|\boldsymbol{J}|$ [Fig. S15, a1-a7], we now also show the current vorticity $(\partial_x J_y - \partial_y J_x)$ [Fig. S15, b1-b7], which clearly delineates to locations of high interface resistance.

2. Following Question #1, it is unclear whether the authors used a diffusive or a hydrodynamic transport regime in the finite element simulations employed to extract the interface resistance. The

modeling seems to assume diffusive behavior, while later the manuscript argues that zero bias and zero magnetic field correspond to a hydrodynamic regime. This discrepancy may significantly affect the extracted interface resistance values. The authors are encouraged to clarify which regime was assumed and justify its validity.

Reply: The interface resistance values were estimated in the diffusive transport regime ($D_\nu = 1$ nm) and kept the same for the hydrodynamic simulation ($D_\nu = 100$ nm). We show below (with point 3) that the best estimate of interface resistance of high R_C region does not depend on the transport regime of graphene.

In response, we added a clarification in the paragraph about the conductivity of the interface region to Section 2.2 of the SI.

3. Continuing from Question #2, in Fig. 4a, the authors attribute the enhanced current in the upper-left graphene channel to hydrodynamic flow at zero magnetic field, transitioning to a diffusive regime at higher fields (e.g., Fig. 3b). However, in Fig. 4d, the current profiles in the lower-right region at zero bias (VBG = 0 V) exhibit no clear difference between zero and high magnetic fields. What causes this discrepancy between the two regions? Does this suggest a significant role of the interface resistance? If so, by what mechanism does the interface resistance lead to such distinct current profiles in the hydrodynamic versus diffusive regimes?

Reply: Yes, we believe that the discrepancy between the upper-left and bottom-right regions is caused by the non-uniformity of the interface resistance (much higher in the upper-left region compared to the lower-right region). This discrepancy can also be seen in Fig. 3b, where the current in the upper-left region shows a field dependence, whereas the current in the lower-right region is mostly unaffected by the magnetic field.

The exact mechanism causing this effect is not easy to pin down due to the complex geometry of our hybrid device. This is why we resort to numerical simulations, which confirm the experimental observation. In broad terms, the high interface resistance in the upper-left region hinders current from entering the metal disc, which effectively leads to a graphene curved channel. Since this channel is relatively narrow (around $0.3\ \mu\mathrm{m}$ that is comparable to D_ν of room-temperature graphene (Ref.4&7)), hydrodynamic effects are expected to play a more prominent role. By contrast, there is no well-defined “channel” in the lower-right region, because the current can pass easily into the metal. Therefore, hydrodynamic effects are much less pronounced.

If the interface resistance suppresses current transfer from graphene into the metal disk in the hydrodynamic regime, then why does such suppression appear to occur at the upper-left region but not at the lower-right region? Is this discrepancy attributable to a difference in interface resistance between the two regions? If so, the authors should specify the approximate range of interface resistance at which it begins to alter the current profiles across the hydrodynamic–diffusive transport crossover, at least based on their simulations.

Reply: The high interface resistance in the upper-left region suppresses current transfer in any regime (hydrodynamic or diffusive). The reason why this suppression only occurs at the upper-left region but not at the lower-right region is that the upper-left region has a much higher interface resistance compared to the lower-right region, as discussed in point 1 above.

Fig. R1 below shows simulated current density maps with varying interface resistance in the upper-left region, for both the diffusive and hydrodynamic regime. The simulations show that once the interface conductance is below ca. 10^{-6} S, current starts flowing through the upper-left portion of the graphene ring in both regimes. Therefore, the interface conductance of the upper-left region in our device must be less than 10^{-6} S. Furthermore, in this range of interface conductance, the difference of current profiles in the upper-left region between hydrodynamic and diffusive regimes become pronounced.

Fig. R2 shows current density linecuts (J_δ) through the device center with a tilt angle $\delta = -32.5°$ (same as the dashed line in Fig. 3b) for quantitative comparison. The figure also clearly shows that the hydrodynamic case is in much better quantitative agreement with the experiment (black crosses) than the diffusive case.

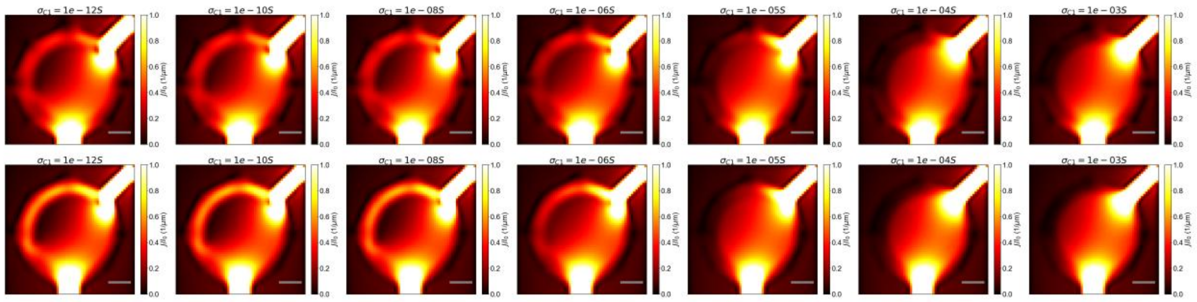

Fig. R1. Supplementary simulation of the 135° contact configuration for varying interface conductivity of high R_C region ($\sigma_{C1} = t/R_{C1}$, where $t = 20$ nm is taken as the width of the graphene-metal interface). Current density maps are shown for values of σ_{C1} between 10^{-12} and 10^{-3} S, corresponding to R_{C1} values between $2 \times 10^{-5}\ \Omega \cdot \mathrm{m}$ and $2 \times 10^{4}\ \Omega \cdot \mathrm{m}$, respectively. The first row assumes a diffusive ($D_\nu = 1$ nm) and the second row a hydrodynamic ($D_\nu = 100$ nm) transport regime, respectively.

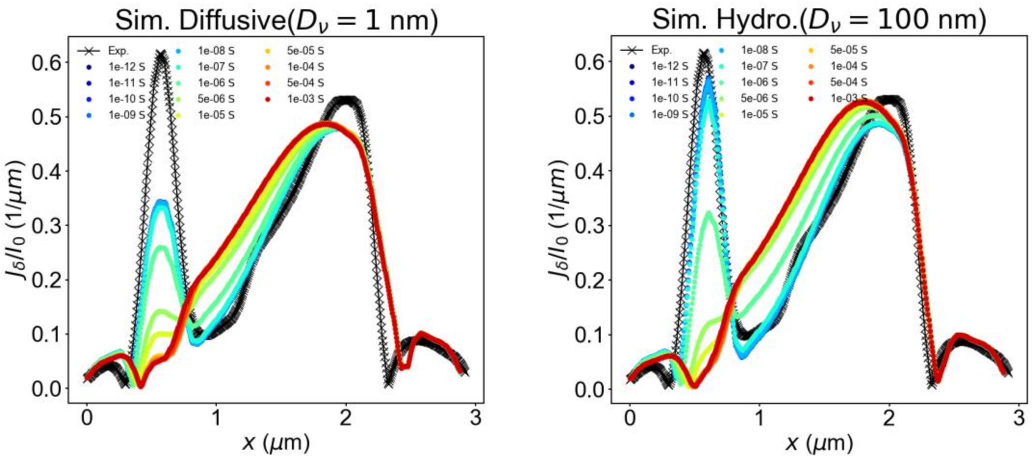

Fig. R2. Line cuts through the plots of Fig. R1 plotting the current density as a function of position. The line cuts were taken along the dashed line in Fig. 3b in the main text. The plots show the comparison between experimental data (black crosses) and simulations (dots with different colors representing different σ_{C1}). The left and right plots correspond to diffusive and hydrodynamic simulations, respectively. Compared to Fig. R1, simulations with more intermediate values of σ_{C1} are also included.

The authors also mention possible degradation of the graphene–metal interface over time in the supplementary materials, which could alter the interface resistance. Did the enhanced current in the upper-left region diminish over time as a result of a reduced interface resistance, or, conversely, did the current in the lower-right region increase due to an increased interface resistance?

Reply: We did not observe that the current in the upper-left region diminished over time, but we did observe (after about half a year) that the current in the lower-right region increased in the graphene channel. We attribute this to an increased interface resistance along the lower-right graphene-metal boundary.

During the ca. 9-months measurement period, the interface between the graphene and the metal disk experienced two degradation events, manifested by an irreversible extension of high interface resistance region along the circular graphene-metal boundary. We accounted for this by modelling the state of the device as s_0, s_1, s_2, see Tables S1 and S2. The degradation events did not occur during measurements, but rather after changes made to the setup, e.g., re-wirebonding of the device. Therefore, we suspect that the degradation was due to some ESD during handling. We also note that even though the degradation of the graphene-metal interface is clearly visible from the magnetometry scans, the two-terminal resistance does not reflect this, which emphasizes the value of local current imaging techniques for revealing microscopic details of the device performance.

In response, we added a paragraph to Section 2.2 in the SI clarifying the degradation of the graphene-metal interfaces in our device.

Overall, a more careful comparison of the current profiles in the two regions around the disk—explicitly distinguishing hydrodynamic vs diffusive transport and clarifying the role of interface resistance in these regimes—would help strengthen the authors' conclusions.

Reply: We thank the Reviewer for the constructive suggestions. We hope that the above explanations and additions to the SI provided some clarity.

(2) Concerning the intrinsic magnetoresistance (MR)
In Fig. 3e, the graphene current (%) at zero fields suddenly drops at higher magnetic fields and then remains nearly constant beyond approximately ±0.1 T. On the other hand, the resistance measurements near CNP in Fig. 4b show a noticeable difference between zero field and ±0.54 T, which the authors interpret as evidence of intrinsic MR.
It would be helpful for the authors to provide resistance data at intermediate magnetic fields (e.g., 0.1 T to 0.5 T) and discuss whether the resistance remains nearly constant in this range, consistent with the current imaging data in Fig. 3e. This comparison would clarify whether the magnetoresistance originates from intrinsic transport properties or could instead be explained by a transition between hydrodynamic and diffusive regimes or by variations in interface resistance.

Reply: The resistance data corresponding to Fig.3e (with Q-V source-drain configuration and at $V_{BG} = 0$) is shown in Fig.1e, which includes the resistance measured at intermediate magnetic fields. These data show that the resistance increase is approximately parabolic with magnetic field, as expected from theory. This increase is in contrast with the graphene current (obtained from current density images) in Fig.3e, which remain approximately constant at higher magnetic fields. Together, the data show that the observed MR is mostly of intrinsic origin, as extrinsic origin would be associated with current re-distribution.

Furthermore, since our observed MR up to ~0.5T is positive, it cannot be explained by a transition from a hydrodynamic to diffusive regime that features a negative MR (Ref. 23). Regarding variations in interface resistance, in our model we assume that they are independent of the external magnetic field so they should not contribute to the MR. Although this assumption may not be strictly true, the measured carrier dependence of resistance (Fig.4b and Fig.S7a) clearly shows a larger MR around charge-neutrality compared to away from charge-neutrality, which suggests that the MR is dominated by the transport properties of graphene instead of the interface.

In response, we clarified in the captions of Fig. 1 and Fig. 3 that the MR curve (Fig. 1e) and imaging data (Fig. 3) were measured concurrently. That is, the resistance data of Fig. 1e was measured with Q-V contact configuration, and each data point corresponds to a magnetometry scan.

Moreover, the cited reference 24 reports a linear MR in this field regime. The present measurements (i.e., no field dependence near CNP based on Fig. 3e) seems to be inconsistent with the reference and wonder if they provide sufficient evidence to claim the intrinsic MR. If the intrinsic MR is indeed the origin, the authors are encouraged to suggest its potential origins more explicitly.

Reply: Ref. 24 reports intrinsic MR in high-mobility graphene, from a parabolic MR in non-quantizing magnetic fields to a linear MR in quantizing magnetic fields. In their anomalously high-mobility sample (>$10\ \mathrm{m}^2\cdot\mathrm{s/V}$ at room temperature), parabolic MR is observed only in small magnetic fields up to about 0.1T, followed by a linear MR at higher magnetic fields. By contrast, in their low-mobility sample (about $0.85\ \mathrm{m}^2\cdot\mathrm{s/V}$), which is presented in Extended Data Fig. 8, parabolic MR persists up to 1T, followed by a sublinear MR at higher fields. Therefore, graphene with a lower mobility exhibits a parabolic MR (at charge neutrality) persisting up to a higher magnetic field.

Since the graphene mobility of our device is estimated to be about $1.4\ \mathrm{m}^2\cdot\mathrm{s/V}$, which is close to the low-mobility sample in Ref. 24, a parabolic MR (instead of a linear MR) is expected below ~0.7T. As shown in Fig. S7c, our device at charge-neutrality indeed exhibits a parabolic MR at least up to ~0.5T (the maximal magnetic field at which we measured), which is consistent with Ref.24.

The origin of the intrinsic MR around graphene charge-neutrality can be explained by the coexistence of electron and hole carriers. As shown in Eq. S7 that is derived from the semi-classical formalism of the two-carrier model, MR is related to the imbalance ($p = \frac{n_e - n_h}{n_e + n_h}$) between electron and hole densities:

$$\mathrm{MR} = \frac{1 - p^2\left(\frac{\mu_B}{\mu_0}\right)^2}{1 + p^2\left(\frac{\mu_B}{\mu_0}\right)^2(\mu_B B_{\mathrm{ext}})^2}(\mu_B B_{\mathrm{ext}})^2.$$

At charge neutrality, $p = 0$ and therefore $MR = (\mu_B B_{\mathrm{ext}})^2$. As one carrier dominates over the other (towards single-carrier regime), $|p|$ increases and therefore MR gradually vanishes, as shown in Fig. 4bc and Fig. S9.

In response, we added a clarification to the manuscript in the last section (“Electron hydrodynamics and single-carrier transport”) that the intrinsic MR is well explained by the two-carrier coexistence.

Minor corrections and suggestions:
1. Replace “change neutral point (CNP)” with “charge neutral point (CNP)”.
2. Page 8, line 5: I suggest to modify the text to “…the Q–V contact configuration (135°)…” for improved clarity.

Reply: We thank the Reviewer for suggestions and pointing out the typo. We have corrected them.

****

Reviewer #2 (Remarks to the Author):

The work of Ding et al. reported the high resolution direct imaging of magnetotransport by using a scanning diamond sensor under magnetic field up to 0.5 T. The results reveals the distribution of electrical current at nanoscale and the properties of a graphene-metal hybrid device. The work shows the potential of solid-state spins to study complex electrical phenomenon.

Reply: We thank the Reviewer for reviewing our work and for the positive assessment of its impact.

There are several comments that should be addressed:
1, Permanent magnets were used this work to produce high magnetic field. How was the inhomogeneity of the magnetic distribution? Did it affect the spin manipulation and sensing in the area of interest? As it is important to make the bias magnetic vector parallel with NV center axis, it will be helpful to provide more details about the adjusting of magnetic field.

Reply: In our setup, the NV center probe and the permanent magnets are fixed during measurements, and only the sample stage is moving. Therefore, the permanent magnetic field at the NV location does not change and the spin manipulation and sensing are not affected by spatial distribution of the bias magnetic field.

For aligning the bias magnetic field with the NV axis, we make use of the known spatial map of magnetic fields produced by our magnet stack. The key is to find the relative location (local coordinates) of NV in the coordinate system of the magnets. To achieve that, we move the magnets to a set of positions and record the corresponding ODMR spectrum, from which the resonance frequencies can be extracted. We then compare these results with the predicted spatial map of resonance frequencies based on the known magnetic field map. By minimizing the difference between the predicted frequencies and measured frequencies, the local coordinates of NV (as free fitting parameters) can be estimated. In general, the more positions where the ODMR is measured, the better the estimation. Based on the obtained local coordinates of NV together with the known magnetic field map of the magnet stack, we can determine positions of the magnet stack that lead to a magnetic field aligned with the NV axis at the NV position.

In Fig. R3, we show the calculated spatial distribution of out-of-plane component (B_z) and in-plane component (B_ρ) of the bias magnetic field produced by our permanent cylindrical magnets, where ρ is the lateral distance from the cylindrical axis and z is the vertical distance from the top surface of the magnet stack. As can be seen, the bias magnetic field close to the cylindrical axis is predominately out-of-plane with $|B_\rho|< 10^{-3}|B_z|$. In addition, the variation of B_z within a $10\mu\text{m}$-radius region is $< 0.01\%$. As the clearance z increases, this variation becomes even smaller. Since the relevant sample area (size of the magnetometry scan) is within $5\mu\text{m} \times 5\mu\text{m}$, it is safe to assume that the sample experiences a uniform bias magnetic field.

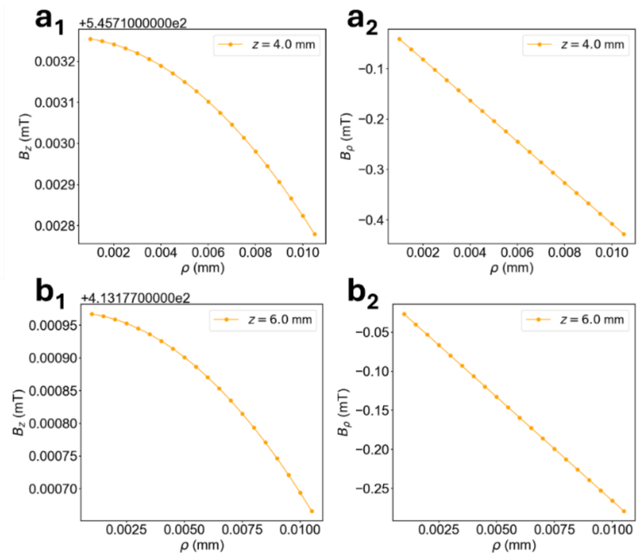

Fig. R3. Simulated magnetic field distribution produced by the permanent magnet stack at a clearance of 4 mm (**a**) and 6 mm (**b**). B_z is the out-of-plane and B_ρ the in-plane component, respectively.

In response, we added a paragraph to the Methods section describing the magnet alignment ("Scanning NV magnetometer setup").

2, In addition, microwave was transmitted through an aluminum wire for NV center spin manipulation. Will the metallic electrodes of hybrid graphene-metal device change the local microwave distribution, and subsequently reduce the spin manipulation fidelity?

Reply: We did not observe any position dependence of the Rabi frequency, indicating that the microwave distribution was uniform across the scan area. This agrees with an early study we performed on a similar graphene device (Fig. 2f in Palm et al., Phys. Rev. Appl. 17, 054008 (2022)). Further, we observe no position dependence of the spin echo contrast, which serves as a measure of the spin manipulation fidelity, see Fig. R4.

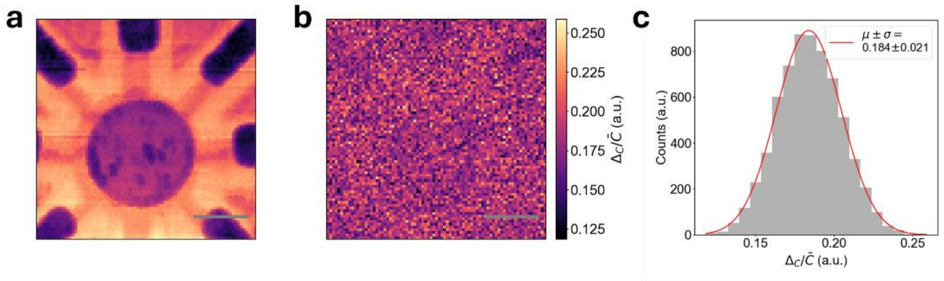

Fig. R4. **a**. AFM image simultaneously acquired with the reference measurement shown in Fig. S5f. **b**. Spin echo contrast map of the reference measurement shown in Fig. S5f. **c.** Histogram obtained from (**b**). The red curve is a Gaussian fit. Scale bar, 1 μm.

3, How was the sensitivity of NV center under high magnetic field?

Reply: The magnetic field sensitivity can be calculated as $\eta_B(\mathrm{T}/\sqrt{\mathrm{Hz}}) = \sigma_B\sqrt{T_{\mathrm{int}}}$,where $\sigma_B(\mathrm{T})$ is the magnetic field uncertainty and $T_{\mathrm{int}}(\mathrm{Hz}^{-1})$ is the integration time. Experimentally, we can estimate σ_B from the standard deviation of the magnetic field image when no current is sent through the device. Fig. R5 below presents such a reference B_{NV} image taken together with the scan presented in

Fig. 4d (at $B_{\text{ext}} = 0.54$ T and $V_{\text{BG}} = 0$) as an example, where $T_{\text{int}} \approx 3.3$ s for this reference experiment at each pixel. Fitting the with a Gaussian, we obtain $\sigma_B \approx 28$ nT, which corresponds to a sensitivity $\eta_B \approx 51\ \text{nT}/\sqrt{\text{Hz}}$.

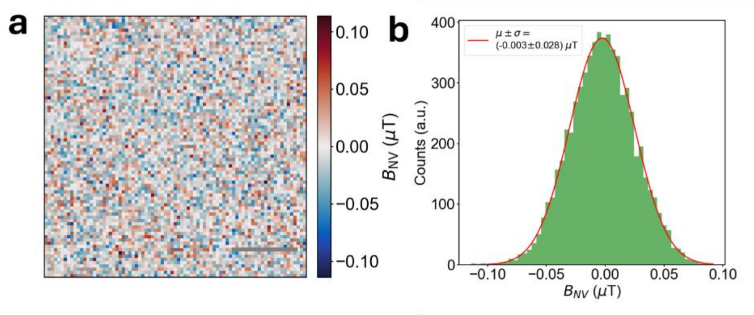

Fig. R5. Reference B_{NV} map (**a**) and corresponding histogram (**b**). The red curve is a Gaussian fit. Scale bar, 1 μm.

In response, we added a paragraph about magnetic field sensitivity to the Methods ("Quantum phase measurement protocol").

4, The current with a frequency of 30 – 300 kHz was detected. Was the frequency kept the same in Fig.1-4? If not, please give the exact value. Will the transport properties of device change with current frequency?

Reply: The frequency of the current is not kept the same for all measurements in Fig.1-4. It is set by the sensing time τ of the spin echo protocol (Fig. S4), which is chosen to optimize the sensitivity and the range of the acquired quantum phase (e.g., for avoiding phase wrapping, see Ref. 6).

In general, within the frequency range mentioned in the text, there is no clear dependence of transport properties on frequency. As a representative example, in Fig. R6 below, we show the normalized B_z images of two magnetometry scans taken under $B_{\text{ext}} = -0.53$ T with different current frequencies f.

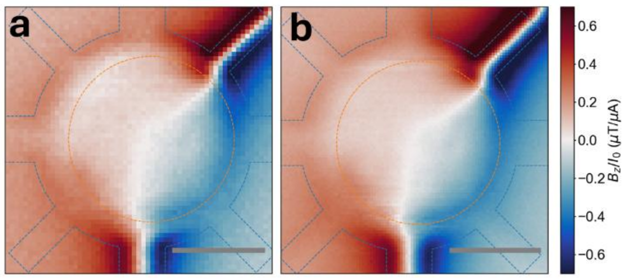

Fig. R6. Normalized B_z maps for two frequencies of the source-drain ac current: **a** (pixel size:50nm):$f = 28.6$ kHz ($\tau = 35$ μs). Simultaneously measured resistance: 25.9 $k\Omega$. **b** (pixel size:30nm): $f = 111.1$ kHz ($\tau = 9$ μs). Simultaneously measured resistance: 26.2 $k\Omega$. Scale bar, 1 μm.

In response, we now state the frequencies used for the scans shown in Fig. 1-4 in Methods section ("Measurement parameters for Figs. 2-4").

5, In Fig 4d, small current leakage was also observed at contacts Q and W, which was absent in the simulation of Fig. S14. And it changed with the magnetic field, while VBG = 0V. Can the author give a brief explanation?

Reply: We thank the Reviewer for the detailed look at our data. In the simulation, we only considered the current leakage at contact S because it is the most prominent one according to our measurements, draining ~25% (28%) of the total current in Fig. 4d at $V_{\mathrm{BG}} = 0$ under low (high) B_{ext}. There are smaller leakage currents at other contacts like Q and W, which we did not include in the model because their magnitudes are relatively minor (e.g., ~8% of the total current at contact Q in Fig.4 d at $V_{\mathrm{BG}} = 0$ under low B_{ext}, and smaller for contact W).

The variation of the leaking current with V_{BG} is due to the associated change of the conductance of graphene. Since graphene has a lower conductance closer to charge-neutrality (near $V_{\mathrm{BG}} \approx 0$), a higher fraction of the current will flow through the leaky contacts.

Next, increasing $|B_{\mathrm{ext}}|$ suppresses hydrodynamic transport and further increases the resistivity of graphene. This results in more current flowing through the lower-right region, increasing the leakage through the lower-right contact W. By contrast, less current is flowing through the upper graphene ring, reducing the leakage through the upper-right contact Q.

We note that other factors not included in the simplest model, such as change of contact resistance on the leakage routes with $|B_{\mathrm{ext}}|$ and small degradation of the leakage contacts, can also influence the magnitude of the (small) leakage currents. Pinpointing their exact origin would require a more systematic investigation. Nevertheless, since these leakage currents are below 10% of the total current, they don't interfere with our main observation and conclusions in Fig. 4.

****
Summary of changes:

Main text:
1. P4, Fig. 1e caption: We adjusted the text to clarify that Fig. 1e and Fig. 3 were measured concurrently.
2. P7, Fig. 3e caption: We likewise adjusted the text to clarify that Fig. 1e and Fig. 3 were measured concurrently.
3. P6, second paragraph: We replaced "change neutrality point" to "charge neutrality point".
4. P9: We extended the paragraph with “Next, to understand…” to clarify Reviewer#1’s questions regarding Fig. 4b.

Methods:
5. P14: We added a paragraph describing the magnet alignment in the "Scanning NV magnetometer setup" section.
6. P15: We added a paragraph about magnetic field sensitivity in the "Quantum phase measurement protocol" section.
7. P19: We added current frequencies used for each dataset in the "Measurement parameters for Figs. 2-4" section.

Supplementary Information:
8. P7-8: We added more details about the graphene-metal interface region, including its conductivity and degradation in Section 2.2.

9. P9-10: We added a subsection "Necessity of including interface resistances" in Section 2.2.
10. We added Fig. S15 (Supplementary data for demonstrating the existence of non-uniform interface resistance along the circular graphene-metal boundary)

Further changes:

11. We made minor changes to main texts, methods, and supplementary information for conciseness and clarification.
12. We added a few references.

Reviewer #1 (Remarks to the Author):

The authors have carefully addressed all of my questions and have reflected them in the main text, figures, and supplementary materials. Therefore, I recommend publication of the manuscript, except for the following issues, which require clarification.

Reply: We thank the Reviewer for their positive feedback and recommendation. We address the questions point-by-point below.

Authors' response to Reviewer #1:
"In response, we add Fig. S15 and Subsection 2.2 ("Necessity of including interface resistances") to the SI. In addition to the current density $|\boldsymbol{J}|$ [Fig. S15, a1-a7], we now also show the current vorticity ($\partial x J y - \partial y J x$) [Fig. S15, b1-b7], which clearly delineates to locations of high interface resistance."
Reviewer #1:
I cannot find the newly added Fig. S15; it appears to be identical to the previous version. I am also confused by the panel labeling (e.g., a1). It is possible that the updated figure did not appear correctly when the file was saved as a PDF. Please double-check this.

Reply: We thank the Reviewer for pointing out the issue. We double-checked that the latest, correct version of the Supplementary Material contains the updated Fig. S15.

Authors' response to Reviewer #1:
"P7, Fig. 3e caption: We likewise adjusted the text to clarify that Fig. 1e and Fig. 3 were measured concurrently."
Reviewer #1:
I can find the corrected statement in the caption of Fig. 1e, but not in the caption of Fig. 3e or Fig. 3. Please double-check this as well.

Reply: We likewise double-checked the main manuscript to make sure that the statement is mentioned in the caption of Fig. 3.

****
Reviewer #2 (Remarks to the Author):

The authors have answered all my comments. I recommend the publication.

Reply: We thank the Reviewer for the positive feedback and recommendation.

****
